# Spatially resolved deconvolution of the fibrotic niche in lung fibrosis

**DOI:** 10.1016/j.celrep.2022.111230

**Published:** 2022-08-16

**Authors:** Michael Eyres, Joseph A. Bell, Elizabeth R. Davies, Aurelie Fabre, Aiman Alzetani, Sanjay Jogai, Ben G. Marshall, David A. Johnston, Zijian Xu, Sophie V. Fletcher, Yihua Wang, Gayle Marshall, Donna E. Davies, Emily Offer, Mark G. Jones

**Affiliations:** 1 Medicines Discovery Catapult, Alderley Park, Cheshire, UK; 2 Clinical and Experimental Sciences, Faculty of Medicine, University of Southampton, Southampton, UK; 3 NIHR Southampton Biomedical Research Centre, University Hospital Southampton, Southampton, UK; 4 Department of Histopathology, St. Vincent’s University Hospital & UCD School of Medicine, University College Dublin, Dublin, Ireland; 5 Biological Sciences, Faculty of Environmental and Life Sciences, University of Southampton, Southampton, UK; 6 University Hospital Southampton, Southampton, UK; 7 Biomedical Imaging Unit, Faculty of Medicine, University of Southampton, Southampton, UK; 8 Institute for Life Sciences, University of Southampton, Southampton, UK

**Keywords:** fibrosis, spatial transcriptomics, cellular deconvolution, lung, alveolar epithelial cell homeostasis

## Abstract

A defining pathological feature of human lung fibrosis is localized tissue heterogeneity, which challenges the interpretation of transcriptomic studies that typically lose spatial information. Here we investigate spatial gene expression in diagnostic tissue using digital profiling technology. We identify distinct, region-specific gene expression signatures as well as shared gene signatures. By integration with single-cell data, we spatially map the cellular composition within and distant from the fibrotic niche, demonstrating discrete changes in homeostatic and pathologic cell populations even in morphologically preserved lung, while through ligand-receptor analysis, we investigate cellular cross-talk within the fibrotic niche. We confirm findings through bioinformatic, tissue, and *in vitro* analyses, identifying that loss of *NFKB inhibitor zeta* in alveolar epithelial cells dysregulates the TGFβ/IL-6 signaling axis, which may impair homeostatic responses to environmental stress. Thus, spatially resolved deconvolution advances understanding of cell composition and microenvironment in human lung fibrogenesis.

## Highlights

• Spatial gene expression profiles for human lung fibrogenesis
• Changes in homeostatic and pathologic cell populations in the fibrotic niche
• Identification of a bone morphogenesis signature within fibroblastic foci
• Loss of alveolar epithelial *NFKBIZ/*IκBζ expression dysregulates the IL-6 axis

## Graphical abstract

**Figure:**
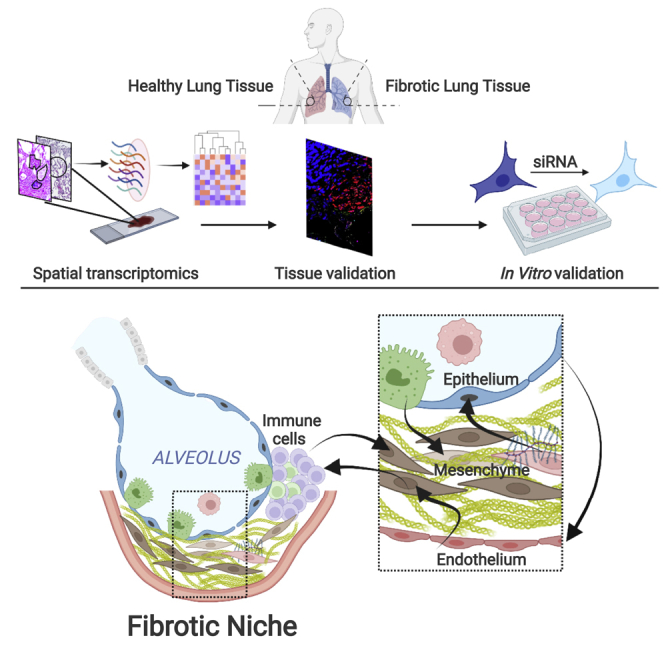

## Teaser

Eyres et al. generate a spatial transcriptomic map of human lung tissue and show how this is perturbed in pulmonary fibrosis. They identify gain and loss of cell populations with evidence of complex cellular cross-talk within the fibrotic microenvironment.

## Introduction

Idiopathic pulmonary fibrosis (IPF) is the prototypic progressive fibrotic lung disease with an average life expectancy of 3–5 years after diagnosis. Treatment options remain limited, so a better understanding of disease pathogenesis remains an area of intense investigation. Idiopathic by definition, the etiology of IPF remains uncertain. Historically it was hypothesized that chronic inflammation resulting from lung injury progressed to tissue damage and fibrosis. In recent years, significant advances have been made in the understanding of human IPF disease pathogenesis. This, together with the recognized absence of benefit, or even potential harm, of immunosuppressive therapies in patients with IPF has supported a shift from this paradigm. Current opinion is that progressive fibrosis is a consequence of multiple interacting genetic and environmental risk factors, with repetitive local micro-injuries to aging alveolar epithelium playing a central role (Richeldi et al., 2017).

Although transcriptomic studies with homogenized IPF lung tissue have provided significant insights into IPF pathogenesis, identifying altered expression of numerous genes and aberrant activation of multiple signaling pathways (DePianto et al., 2015; Yang et al., 2013), the core determinants underlying the initiation and progression of fibrosis remain poorly understood (Mehal et al., 2011; Selman et al., 2008; Selman and Pardo, 2002; Vukmirovic and Kaminski, 2018). Disease heterogeneity within the lung poses a particular challenge to interpretation of such transcriptomic studies in IPF. Tissue shows areas of marked fibrosis adjacent to normal appearing lung as well as architectural distortion and the presence of fibroblastic foci, enigmatic regions pathognomonic of the disease, representing sites of active fibrogenesis (King et al., 2017; Raghu et al., 2006; Richeldi et al., 2017).

Analysis of human IPF pathogenesis studies has focused on mesenchymal and epithelial cell populations. Although dysregulation of multiple cell types has been proposed, the cross-talk between cell populations remains poorly understood. In addition, the presence of aggregates of infiltrating immune cells close to (but not always within) sites of active fibrosis is well described (Nuovo et al., 2012). These immune cells appear unable to penetrate the stroma of fibroblastic foci in a phenomenon similar to that observed in some fibrotic cancers, for example pancreatic tumors (Liu et al., 2021a). In the latter setting, there is increasing evidence that the cross-talk between the tumor cells, cancer-associated fibroblasts, and immune cells may lead to immune suppression and contribute to determine tumor progression (Seager et al., 2017). Thus, in human lung fibrosis, there is a need to better understand the interplay among subpopulations of mesenchymal cells, epithelial cells, immune cells, and extracellular matrix components that cooperatively form the fibrosis-specific microenvironment (i.e., the fibrotic niche).

The recent application of single-cell RNA sequencing (scRNA-seq) approaches to IPF tissue has provided understanding of diverse aberrant cell populations. However a limitation of scRNA-seq is that it does not permit spatial characterization of sequenced cells or the interactions between regions (Adams et al., 2020; Morse et al., 2019; Peyser et al., 2019; Reyfman et al., 2019; Tsukui et al., 2020; Xu et al., 2016), and both homogenized bulk IPF lung tissue and scRNA-seq studies have typically been performed upon end-stage fibrotic lung tissue obtained at time of lung transplantation, which may have less relevance for understanding of early disease processes. Spatial transcriptomics provides the capability to circumvent such issues by generating gene expression data for specific, well-defined regions of interest (ROIs) within tissue sections. The recently established digital spatial profiling (DSP) methodology enables quantitative, multiplex analysis of mRNAs in spatially defined regions within formalin-fixed paraffin-embedded tissues (Merritt et al., 2020), thus offering the possibility to investigate region-specific gene expression profiles in tissue obtained at time of diagnosis.

Here, we investigated spatial gene expression in diagnostic IPF lung tissue using DSP technology and confirmed our findings through bioinformatic, tissue, and *in vitro* analyses. By integration with single-cell data, we spatially mapped the cellular composition within and distant from the fibrotic niche, and we studied cellular cross-talk by performing an analysis of ligand-receptor interactions. Together these approaches demonstrate spatially resolved changes in homeostatic and pathologic cell populations and reduced inflammatory gene expression signatures including decreased innate immunity, and they identify that loss of *NFKB inhibitor zeta* (*NFKBIZ*) in alveolar epithelial cells dysregulates the TGFβ/IL-6 signaling axis, which may impair homeostatic responses to environmental stress in early disease.

## Results

### Digital spatial gene expression profiling of human lung fibrosis tissue

**Figure 1:**
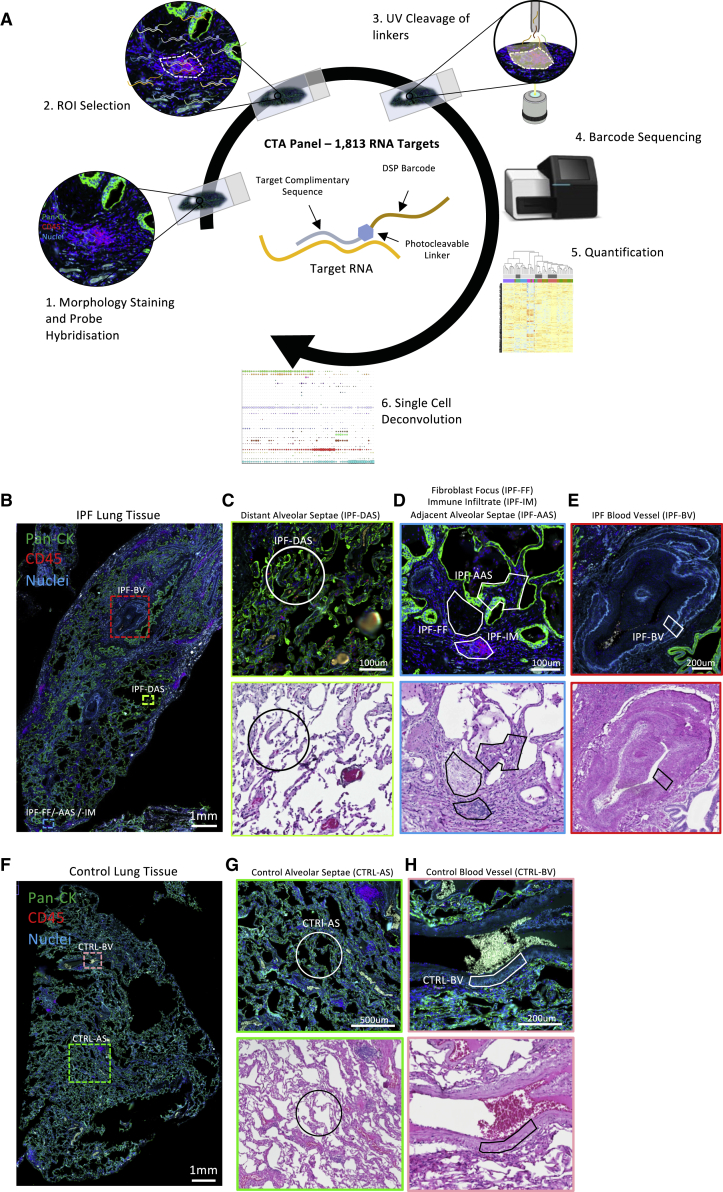
Overview of study and analytical workflow pathway (A) Schematic showing workflow. (B) Overview of IPF tissue stained for pan-cytokeratin, CD45, and nuclei with areas containing regions of interest highlighted. (C–E) Insets from (B) showing different ROIs. Border colors denote area of (B) taken from, with subsequent hematoxylin and eosin (H&E) staining of the tissue section used for DSP. (F) Control lung tissue with areas containing ROIs highlighted. (G and H) Insets from (F) showing ROIs from control lung tissue, with corresponding H&E staining. Scale bars (B and F), 1 mm. Scale bars (C and D), 100 μm. Scale bars (E and H), 200 μm. Scale bar (G), 500 μm.

We characterized the mRNA expression within 66 morphologically distinct ROIs (Figure 1) in diagnostic human IPF lung tissue and control lung tissue using DSP with the Nanostring GeoMx platform (Figure 1A). The tissue was interrogated using the GeoMx Cancer Transcriptome Atlas (CTA) mRNA assay of 1,813 unique genes that encompasses cell biology, metabolic, microenvironment, and immune response pathways. As DSP is non-destructive, following GeoMx analysis, hematoxylin and eosin (H&E) staining was performed on the same tissue sections, and region selection was reviewed by a histopathologist. Following this review, six ROIs were excluded, so 60 ROIs were analyzed (Table S1 and Table S2). ROIs analyzed were fibroblastic foci (IPF-FF, n = 10) and their adjacent alveolar septae (IPF-AAS, n = 10), nearby immune infiltrates identified by CD45+ staining (IPF-IM, n = 6), morphologically preserved alveolar septae distant from fibroblastic foci (IPF-DAS, n = 10), and blood vessel walls containing smooth muscle (IPF-BV, n = 6) (Figures 1B–1E). In healthy control lung tissue, selected ROIs were alveolar septae (CTRL-AS, n = 12) and blood vessel walls containing smooth muscle (CTRL-BV, n = 6) (Figures 1F–1H). Applying an expression cutoff of 5% (i.e., genes expressed in >5% of samples), a total of 1,113 genes out of 1,813 in the CTA were selected for further analysis.

### Distinct gene expression signatures in spatially resolved regions of lung tissue

**Figure 2:**
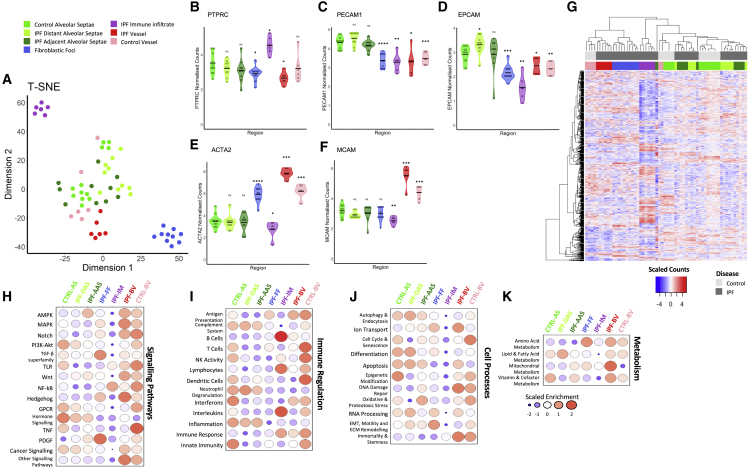
Distinct gene expression signatures in spatially resolved regions of lung tissue (A) t-Stochastic nearest neighbor embedding (t-SNE) dimensional reduction plot of each region of interest (ROI). (B–F) Violin plots of gene expression values of cell population marker genes. Statistical comparisons are relative to control alveolar septae. ^∗^p < 0.05, ^∗∗^p < 0.01, ^∗∗∗^p < 0.001, ^∗∗∗∗^p < 0.0001 by Wilcoxon test with Benjamini-Hochberg multiple test correction. (G) Heatmap showing differentially expressed genes (as measured by a Kruskal-Wallis test; p < 0.05) across dataset, showing clustering of different ROI groups. (H–K) Bubble plots for control and IPF ROIs showing scaled enrichment scores for (H) signaling pathways gene sets, (I) immune regulation gene sets, (J) cell processes gene sets, and (K) metabolism gene sets.

We first determined biologically plausible clustering of ROIs when visualized by t-stochastic nearest neighbor embedding (t-SNE) dimensional reduction plot (Figure 2A) and a Pearson correlation heatmap (Figure S1), identifying separation of different ROIs, with this most evident for immune infiltrates and fibroblastic foci. We confirmed enrichment of expected marker genes within ROIs (Figures 2B–2F) as well as differentially expressed genes across the dataset (Figure 2G). The highest expression of *PTPRC* (CD45), an immune marker, was identified in the immune infiltrate, *PECAM1* and *EPCAM* (endothelial and epithelial markers, respectively) within alveolar regions, *ACTA2* (alpha smooth muscle actin, a smooth muscle and myofibroblast marker) in the vessel walls and fibroblastic foci, and *MCAM* (expressed by cells constituting blood vessels) within blood vessel walls.

To visualize whole signaling pathways and cell processes that were activated or repressed within different regions of tissue, we graphed (Figures 2H–2K) normalized gene expression values grouped by gene sets (Table S3). Comparison of control and IPF alveolar septae ROIs identified enrichment of Wnt and PDGF signaling in IPF alveolar septae, whereas there was suppression of TNF signaling (Figure 2H) and interferons (Figure 2I). Comparison of spatially related IPF ROIs (IPF adjacent alveolar septae, immune infiltrate, and fibroblastic foci) showed enrichment of cell processes involving EMT, ECM, and motility in fibroblastic foci and adjacent alveolar septae (Figure 2J), with signaling pathways including PDGF and TGFβ superfamily signaling enriched in fibroblast foci (Figure 2H). Within the vessels, ion transport and mitochondrial metabolism were increased (Figures 2J and 2K), while TNF signaling was decreased (Figure 2H).

Together these data confirm successful GeoMx DSP, identifying biologically plausible differential expression changes within distinct ROIs in normal and fibrotic lung tissue.

### Identification of spatially resolved changes in homeostatic and pathologic cell populations in lung fibrosis

**Figure 3:**
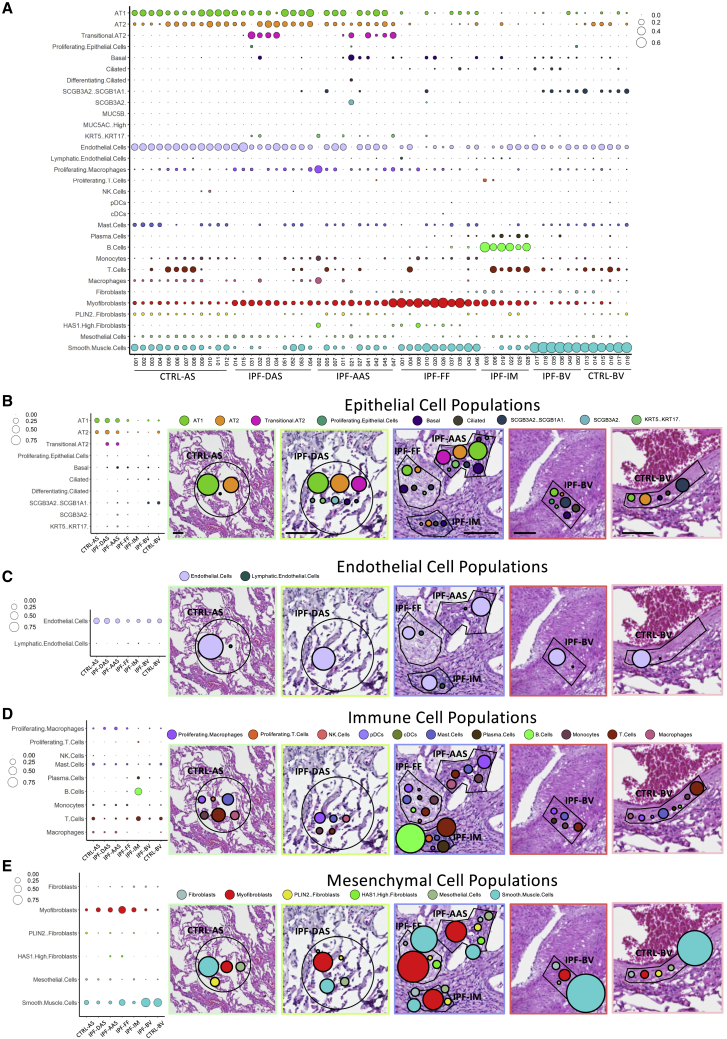
Spatially resolved cell populations in lung fibrosis and control lung tissue (A) Bubble plot showing proportion of cell types in each ROI calculated by spatial deconvolution. (B–E) Proportions of epithelial, endothelial, immune, and mesenchymal cell populations in different ROIs. Representative H&E images are higher magnification of regions visualized in Figure 1. Scale bars, 100 μM.

To investigate the cellular composition within each spatially defined ROI, we used the spatial deconvolution methodology (Danaher et al., 2020), applying a signature matrix, generated from a single-cell RNAseq dataset of 31 cell types derived from healthy and IPF lung tissue (Habermann et al., 2020) (Figure 3A). Alveolar septae contained predominantly ATI, ATII, and endothelial cells (Figures 3B and 3C), and immune infiltrates contained an array of different immune cell types, including B, T, and plasma cells (Figure 3D), while fibroblastic foci contained multiple mesenchymal cell types (Figure 3E).

When comparing the cellular composition of alveolar septae in control tissue with those distant from and adjacent to sites of fibrosis, the proportion of alveolar type I and II cells was not substantially different (Figures 3B, S2A, and S2B). However, basal cells and the recently proposed pathologic KRT5^−^/KRT17^+^ cell population were only identified within IPF tissue, and their proportion increased from distant to adjacent alveolar septae (Figures 3B, S2C, and S2D).

We also observed an increase in the proportion of proliferating macrophages from control to distant alveolar septae to those adjacent to sites of fibrosis (Figures 3D and S2E), whereas there was a reduction in endothelial cells in IPF alveolar septae with the reduction being greatest in septae close to fibroblastic foci (Figures 3C and S2F). This might be explained by a decrease in expression of the endothelial mitogens, *EDN1* and *VEGF* isoforms in IPF alveolar septae (Figures S3A–S3D).

In contrast to the reduction identified in endothelial cells, there was a stepwise increase in myofibroblasts from healthy control alveolar septae to IPF distant alveolar septae to adjacent alveolar septae (Figures 3E and S2G). Notably, *HAS1*^*hi*^ fibroblasts (Figures 3E and S2H), a recently proposed pathologic IPF fibroblast subtype (Habermann et al., 2020), were also identified within adjacent alveolar septae and fibroblastic foci, while these cells were absent from all other areas. Conversely, PLIN2+ fibroblasts, a suggested homeostatic lipofibroblast-like cell type (Habermann et al., 2020), were present within control and IPF alveolar septae while being poorly represented in fibroblastic foci and IPF blood vessels (Figures 3E and S2I). As expected, smooth muscle cells were the dominant cell type within blood vessel walls (Figures 3E and S2J).

Together these data identify spatially discrete changes in homeostatic and pathologic cell populations in lung fibrosis and suggest that pathogenetic mechanisms may be active, even in morphologically preserved alveolar septae within IPF lung tissue.

### Enrichment of a bone morphogenesis signature within fibroblastic foci

**Figure 4:**
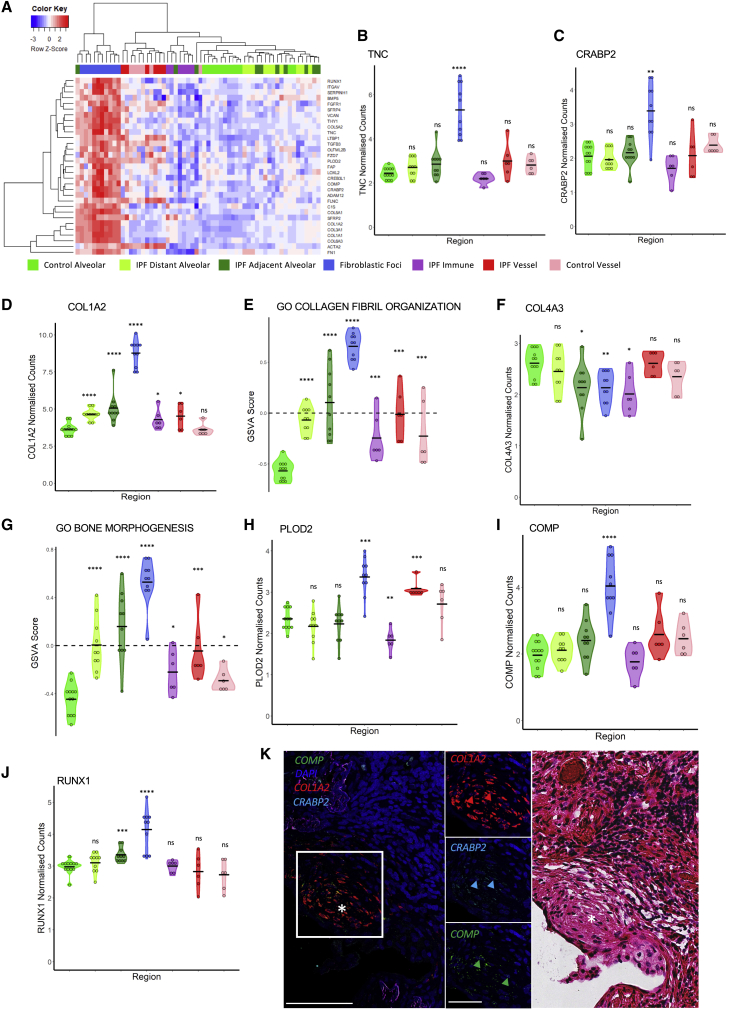
Gene expression within fibroblast foci (A) Heatmap showing top differentially expressed genes between IPF fibroblastic foci and all other ROIs. (B) *TNC* expression. (C) *CRABP2* expression. (D) *COL1A2* expression. (E) *COMP* expression. (F) Collagen fibril organization GSVA scores. (G) *COL4A3* expression. (H) Bone morphogenesis GSVA scores. (I) *PLOD2* expression. (J) *RUNX1* expression. Statistical comparisons are relative to control alveolar septae. ^∗^p < 0.05, ^∗∗^p < 0.01, ^∗∗∗^p < 0.001, ^∗∗∗∗^p < 0.0001 by Wilcoxon test with Benjamini-Hochberg multiple test correction. (K) Representative multiplexed RNA *in situ* hybridization for *COMP, COL1A2*, and *CRABP2* expression with a fibroblastic focus identified by ^∗^, with the corresponding fibroblast focus (^∗^) identified by H&E staining on the same tissue section. Scale bar, 100 μm. Inset scale bar, 50 μm.

We next studied the differentially expressed genes enriched within fibroblastic foci (Figure 4A). Tenascin C (*TNC*), a gene encoding an ECM glycoprotein that is increased in response to tissue injury, was upregulated in fibroblastic foci, but not within the alveolar septae ROIs, (Figure 4B) as well as *CRABP2*, a retinol-binding protein whose expression has recently been associated with IPF disease progression (Figure 4C) (Ghandikota et al., 2022). We also noted that all ROIs in IPF showed a generalized increase in the Hallmark glycolysis gene set (Figure S3I), and that this effect was greatest in fibroblastic foci that contained the largest proportion of myofibroblasts, cells that are known to switch to aerobic glycolysis in response to TGF-β (Xie et al., 2015). We identified a significant increase in multiple fibrillar collagen genes including *COL1A2* in fibroblastic foci, as well as an enrichment of the collagen fibril organization gene set (Figures 4D and 4E). In contrast, *COL4A3*, a basal lamina-associated collagen, was not increased in IPF fibroblastic foci (Figure 4F). Fibrillar collagen genes were also increased in IPF alveolar septae: this expression progressively increased from morphologically preserved alveolar septae to those adjacent to fibroblastic foci. This observation is in keeping with the progressive increase in mesenchymal cell proportions identified in our spatial deconvolution analysis.

In addition to collagen fibril assembly, gene set variation analysis also identified a progressive upregulation of genes associated with bone morphogenesis (Figure 4G), with the expression of *PLOD2,* which encodes the lysyl hydroxylase 2 (LH2) enzyme required for pyridinium-type collagen cross-linking that predominates within bone (Qi and Xu, 2018), also upregulated in fibroblastic foci (Figure 4H), as well as expression of *COMP* that encodes cartilage oligomeric matrix protein and is primarily expressed within cartilage (Song et al., 2003) (Figure 4I). Further supporting this bone-type pathology is the upregulation of Runt-related transcription factor 1 (*RUNX1*), a transcription factor associated with increased expression of factors associated with osteogenesis (Tang et al., 2021) (Figure 4J). Although *RUNX1* is also associated with control of blood cell development (Imperato et al., 2015, p. 1), we noted a relative paucity of hematopoietic cells in fibroblastic foci. Using RNAscope *in situ* hybridization (RNA-ISH), we confirmed high expression of *COL1A2*, *COMP*, and *CRABP2* within fibroblast foci (Figure 4K), with immunofluorescence staining identifying the expression of COMP and PLOD2 within fibroblast foci (Figure S3J).

### Ligand-receptor interactions within the fibrotic niche

**Figure 5:**
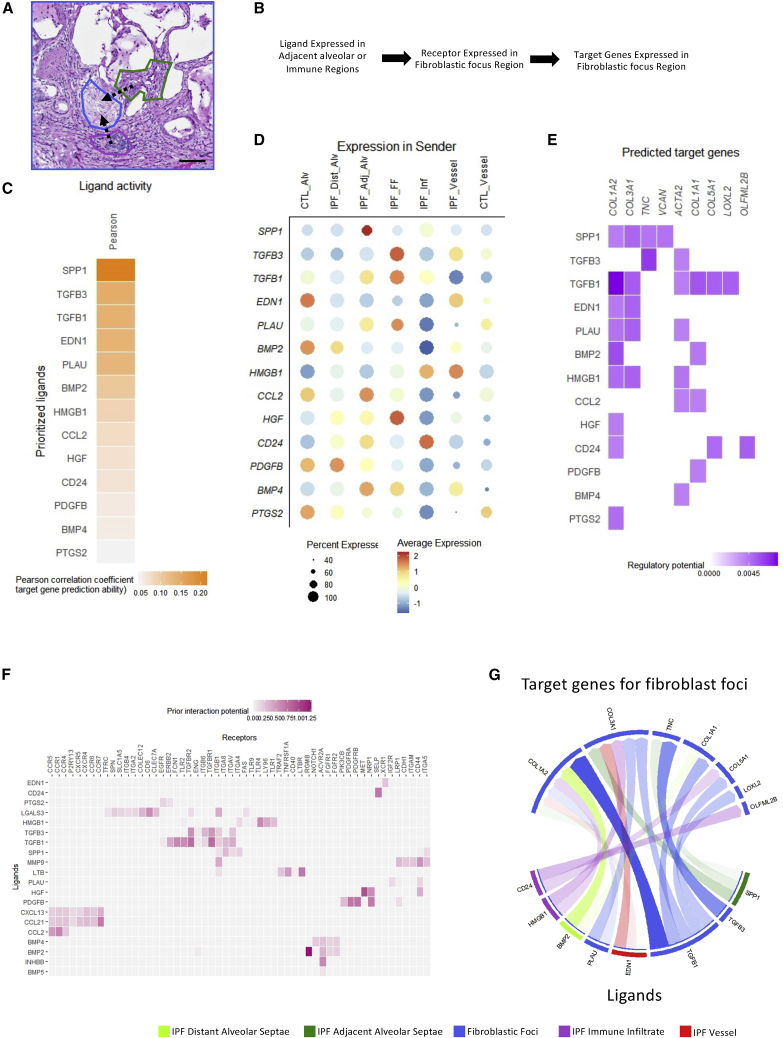
Ligand-receptor interactions within the fibrotic niche (A) Representative H&E stained IPF lung tissue section of a fibroblast focus, immune infiltrate, and adjacent alveolar septae within the fibrotic niche following digital spatial profiling. Scale bar, 100 μm. (B) Schematic of NicheNet workflow. Nichenet predicts communications based on ligand expression in sender regions, receptor expression in receiver regions, and signaling within the sender regions. (C) Pearson correlation coefficients of ligands and target genes in adjacent ROIs. (D) Dot blot showing mean ligand expression in different ROIs. (E) Regulatory potential of predicted target genes in IPF fibroblastic foci. (F) Receptors expressed within fibrotic foci regions that can potentially bind to ligands found in (C). Heatmap shows the regulatory potential for ligand-receptor pairs based on prior interaction knowledge. (G) Results from (C)–(E) summarized in a circus plot. Arrow transparency indicates regulatory potential between ligand and target gene. Arrows are colored depending on the region in which the ligand is most highly expressed.

DSP enables the quantification of the gene transcripts within distinct adjacent regions in tissue that may contain different cell types, thereby providing the possibility to investigate spatially resolved intercellular communication within the fibrotic niche. To determine how alveolar septae and immune infiltrates may signal to fibroblastic foci, we therefore performed an analysis of ligand-receptor interactions. We applied NicheNet (Browaeys et al., 2020), a computational method that predicts ligand-receptor interactions based on induction of downstream target genes (Figures 5A and 5B). This identified the potential for signaling from adjacent regions to upregulate genes expressed in fibroblastic foci, with predicted signaling from adjacent alveolar septae via BMP4, CCL2, CD24, HGF, SPP1, PLAU, and TGFβ1, and signaling from immune infiltrates via CD24, HMGB1, SPP1, and TGFβ1 (Figures 5C and 5D). Predicted target genes included numerous extracellular matrix-related genes driven by expression of multiple receptors present in fibroblastic foci (Figures 5E–5G). We additionally performed *in silico* ligand-receptor analysis using Cellinker (Zhang et al., 2021), confirming the potential for signaling from adjacent regions to fibroblast foci including via HMGB1, PLAU, and TGFβ1 (Figure S4).

SPP1 (osteopontin) was among the most highly expressed ligands in adjacent alveolar septae, whereas HMGB1 and CD24 were among the most highly expressed ligands in immune infiltrates. While SPP1 has been reported to be expressed in epithelial cells (Ali et al., 2019), it is also expressed in proliferating macrophages (Morse et al., 2019), a cell population that we identified in the adjacent alveolar septae using spatial deconvolution analysis (Figure 3D). HMGB1 signaling from immune infiltrates to fibroblastic foci could contribute to the increased expression of fibrillar collagen and alpha smooth muscle actin genes in the fibroblastic foci, a process previously identified in skin fibrosis (Lee et al., 2018). Furthermore, HMGB1 has also been identified as a downstream effector of SPP1 signaling in fibrogenic liver injury (Arriazu et al., 2017), highlighting the potential of distinct cell types within the fibrotic niche to co-ordinately drive a fibrotic response.

### Innate and inflammatory signatures are dysregulated across tissue compartments in lung fibrosis

**Figure 6:**
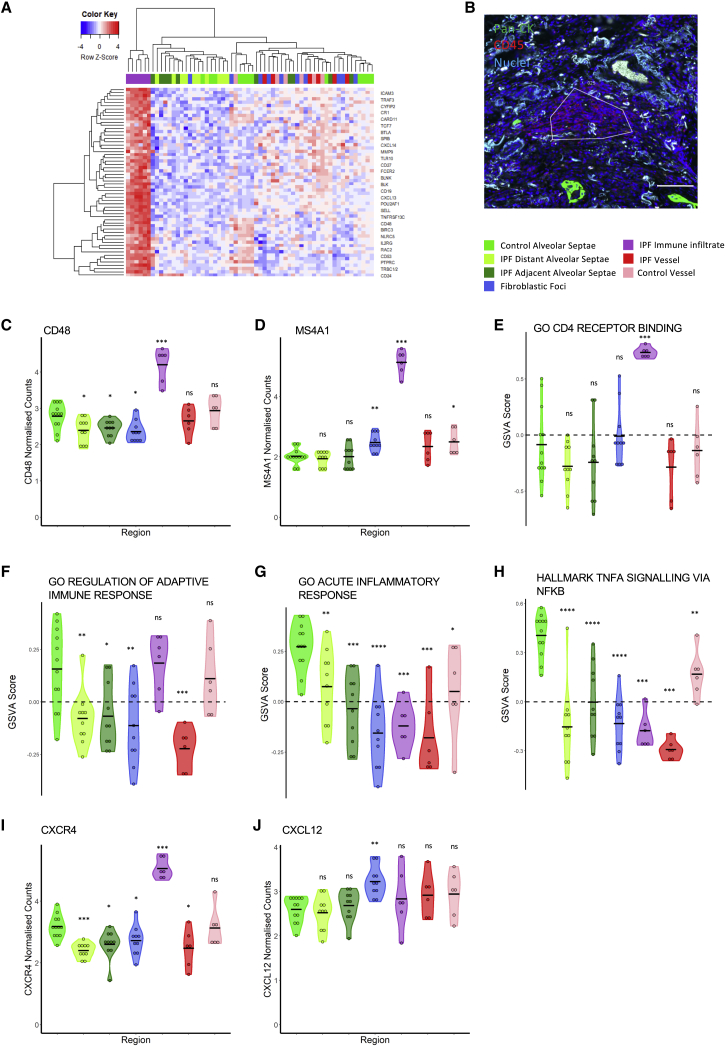
Gene expression within immune infiltrates (A) Heatmap showing top differentially expressed genes between IPF immune infiltrates and all other ROIs. (B) Immunofluoresent staining (pan-cytokeratin, CD45, and nuclei) of IPF tissue with an immune infiltrate ROI highlighted. Scale bar, 100 μm. (C and D) Gene expression in ROIs for *CD48* (C) and *MS4A1* (D). (E–H) Gene set variation analysis (GSVA) scores for ROIs for GO CD4 receptor binding (E), GO regulation of adaptive immune response (F), GO acute inflammatory response (G), and Hallmark TNFA signaling via NFKB (H). (I–J) Expression in ROIs for *CXCR4* (I) and *CXCL12* (J). Statistical comparisons are relative to control alveolar septae. ^∗^p < 0.05, ^∗∗^p < 0.01, ^∗∗∗^p < 0.001, ^∗∗∗∗^p < 0.0001 by Wilcoxon test with Benjamini-Hochberg multiple test correction.

We next investigated immune and inflammatory gene expression signatures. Initially, IPF-IM ROIs were interrogated and differentially expressed genes in these ROIs compared with all others were identified (Figures 6A and 6B). The IPF-IM ROIs show a clear lymphocyte gene expression signature, including several canonical B cell markers such as *CD48* and *MS4A1* (membrane-spanning 4A [CD20]) (Figures 6C and 6D). Gene set variation analysis (GSVA) of the IPF-IM ROIs also confirmed the presence of T cells, with the CD4 receptor binding gene ontology (GO) term enriched in these ROIs (Figure 6E). While GSVA identified an adaptive immune response gene signature in IPF immune infiltrate ROIs, this signature was decreased in other IPF ROIs compared with control ROIs (Figure 6F). Furthermore, there was a downregulation of the acute inflammatory response genes and TNFA signaling via NF-κB gene sets in all fibrotic tissue ROIs when compared with healthy control ROIs (Figures 6G and 6H).

CXCR4, the receptor for the homeostatic chemokine CXCL12, was among the most significantly upregulated genes in IPF-IM ROIs (Figure 6G and 6H). In many solid cancers, expression of CXCL12 by cancer-associated fibroblasts (CAFs) leads to the exclusion of T cells, and we also found that fibroblastic foci were the main source of CXCL12 expression (Figure 6J), suggesting that this chemokine may function to exclude immune cells from the fibrotic niche in lung fibrosis. Using independent single-cell RNA sequencing data, we confirmed expression of CXCR4 in lymphocytes and myeloid cells (Figures S5A–S5C), while CXCL12 was expressed by mesenchymal cells (Figures S5D–S5F), consistent with a recent report identifying an increase in CD45^+^CXCR4^+^ cells within IPF tissue with strong CXCL12 expression within fibroblast foci (Jaffar et al., 2020).

Comparison of the gene signatures associated with IPF-BV compared with CTRL-BV ROI identified a number of genes and gene sets with differential expression, with latent TGFβ binding protein 1 (LTBP1) and IL11 receptor alpha (IL11RA) being upregulated in IPF blood vessels compared with control vessels (Figures S3E and S3F). In contrast, JUNB expression was downregulated in IPF-BV ROIs compared with CTRl-BV (Figure S3G). This was also accompanied by a downregulation of the Hallmark TNFα signaling via NF-κB gene set (Figure S3H).

Together these data suggest that the IPF immune infiltrates consist predominantly of adaptive immune cells, especially B and T cells, consistent with the spatial deconvolution analysis and that a CXCL12/CXCR4 axis may contribute to immunosuppression around the fibrotic niche. There was also an overall decrease in inflammatory gene sets in IPF lung tissue compared with normal lung, whereas IL11RA, a component of IL11/IL11RA/IL6ST signaling complex that is involved in the control of proliferation and/or differentiation of skeletogenic progenitor or other mesenchymal cells, was increased in IPF vascular smooth muscle.

### Suppression of type 1 interferon responses

**Figure 7:**
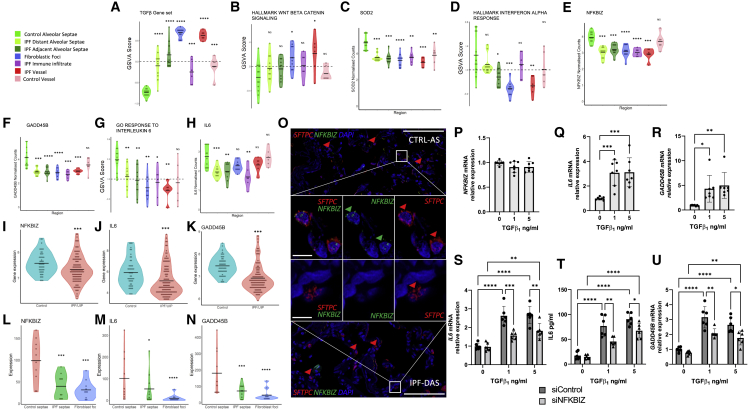
Loss of NFKBIZ dysregulates the IL-6 axis (A and B) GSVA scores for TGFβ or WNT signaling as indicated for different ROIs. (C) Expression in ROIs for *SOD2*. (D) GSVA scores for different ROIs for hallmark interferon alpha response. (E and F) Expression in ROIs for *NFKBIZ* (E) and *GADD45B* (F). (G) GSVA scores for different ROIs for GOresponse to interleukin 6. (H) Expression in ROIs for *IL6.* (I–K). Expression of *NFKBIZ* (I), *IL6* (J), and *GADD45B* (K) in GSE32537. (L–N) Expression of *NFKBIZ* (L), *IL6* (M), and *GADD45*B (N) in GSE169500. Statistical comparisons are relative to control alveolar septae. ^∗^p < 0.05, ^∗∗^p < 0.01, ^∗∗∗^p < 0.001, ^∗∗∗∗^p < 0.0001 by Wilcoxon test with Benjamini-Hochberg multiple test correction. (O) Representative multiplexed RNA *in situ* hybridization for *SFTPC* and *NFKBIZ* expression in control alveolar septae and IPF distal alveolar septae. Scale bar, 100 μm; inset scale bar, 10 μm. (P–R) Type 2 alveolar epithelial cells were treated with TGFβ or vehicle control as indicated for 24 hr. Relative gene expression of *NFKBIZ* (P), *IL6* (Q), and *GADD45B* (R) determined by qRT-PCR and analyzed using ΔΔCt method. Data are mean ± SD; n = 7 across three independent experiments. ^∗^p < 0.05, ^∗∗^p < 0.01, ^∗∗∗^p < 0.001 by two-way ANOVA with Tukey’s multiple comparison test. (S–U) Type 2 alveolar epithelial cells were transfected with *NFKBIZ* targeting siRNA or control siRNA for 48 hr followed by treatment for 24 hr with TGFβ or vehicle control as indicated. (S) Relative gene expression of *IL6* measured as in (Q). (T) IL-6 protein in conditioned media quantified by ELISA. (U) Relative gene expression of *GADD45B* measured as in (T). Data are mean ± SD; n = 6 across three independent experiments. ^∗^p < 0.05, ^∗∗^p < 0.01, ^∗∗∗^p < 0.001, ^∗∗∗∗^p < 0.0001 by two-way ANOVA with Tukey’s multiple comparison test.

Comparison of alveolar septae regions identified that there was a progressive increase in expression of gene sets associated with TGFβ and Wnt signaling from control alveolar septae to distant IPF alveolar septae to IPF alveolar septae adjacent to fibrotic lesions (Figures 7A and 7B) in addition to increased expression of fibrillar collagen genes (Figure 5 and Table S4A). There was also a significant decrease in the expression of the antioxidant enzyme superoxide dismutase 2 (SOD2), which we confirmed with RNA-ISH suggesting lack of ability of IPF tissue to respond to reactive oxygen species generation (Figures 7C and S7A), as well as genes associated with the interferon alpha response (Figure 7D).

We hypothesized that the suppression in type I IFN-regulated gene expression could be explained by the identified increased expression of TGFβ signaling pathways, as we and others have previously reported that TGFβ suppresses type I IFN-regulated gene expression in airway epithelium (Bedke et al., 2012; Denney et al., 2018). To further investigate this potential mechanism, we treated alveolar type 2 cells with TGFβ in the absence or presence of the synthetic TLR3 agonist, polyinosinic:polycytidylic acid (poly I:C), to mimic viral infection. We identified that TGFβ suppressed *IFNB1* expression as well as the interferon inducible genes, *MX1* and *viperin* (Figures S6A–S6C), consistent with the potential for TGF-β to suppress the innate immune response to viral infection.

### Loss of *NFKBIZ* dysregulates the IL-6 axis

We also identified downregulation of *NFKBIZ* and *GADD45B* within IPF alveolar septae (Figures 7E and 7F). These are key components of the interleukin 6 (IL6) signaling pathway (Liebermann and Hoffman, 2002; Yamamoto et al., 2004), and correspondingly we found that GO response to IL6 was downregulated in IPF ROIs (Figure 7G), as well as IL6 expression itself (Figure 7H). We confirmed suppression of these genes in a published bulk microarray dataset comparing IPF and normal lung tissue (Figures 7I–7K). Reduced NFKBIZ expression in IPF alveolar septae was further validated by interrogation of a publicly available laser-capture-microdissection RNA-seq dataset of IPF alveolar septae, confirming a reduction in the expression of *NFKBIZ, IL6* and *GADD45B* in IPF alveolar septae compared with control tissue (Figures 7L–7N). While the protein product of *NFKBIZ*, IκBζ, can inhibit the activity of NF-κB, it can also activate the transcription of a set of genes associated with the overactivation of the immune system in psoriasis (Bertelsen et al., 2017). Consistent with loss of *NFKBIZ*, there was decreased expression of *CCL20*, *CHI3L1, IL8*, and *DEFB4* within IPF alveolar septae (Figures S6D–S6G).

Analyzing publicly available data within the Human Protein Atlas (Uhlén et al., 2015), we confirmed basal *NFKBIZ* gene expression within lung cell populations, especially alveolar type 2 cells and granulocytes (Figure S7B), as well as nuclear localization of IκBζ within alveolar epithelial cells in healthy human lung tissue (Figure S7C). We therefore performed immunostaining of control and IPF lung tissue, identifying IκBζ expression within control alveolar epithelial cells and that this was reduced within IPF alveolar septae (Figure S7D), and confirming through RNA-ISH that *NFKBIZ* was expressed within alveolar type 2 cells identified by the cell marker surfactant protein C (SFTPC) (Figure 7O).

*NFKBIZ* is a primary response gene, induced rapidly in innate immune cells such as monocytes and macrophages in response to pathogen-associated molecular patterns such as the TLR4 agonist lipopolysaccharide (LPS) (Eto et al., 2003). However *NFKBIZ* has also been shown to have cell-specific roles (Ahn et al., 2019; Hörber et al., 2016; Miyake et al., 2010; Touma et al., 2011), and to our knowledge, basal expression of *NFKBIZ* in alveolar epithelial cells and the consequences of reduced expression upon alveolar homeostasis have not previously been investigated.

Therefore, to investigate the impact of loss of *NFKBIZ* on responses of alveolar type 2 cells, we used siRNA knockdown. IL-6 can be induced by proinflammatory cytokines, as well as by TGFβ. Given that we observed downregulation of the acute inflammatory response genes in all fibrotic tissue ROIs whereas TGFβ signaling was increased, we elected to investigate the importance of *NFKBIZ* for TGFβ-induced IL-6 expression. *NFKBIZ* was constitutively expressed in ATII epithelial cells *in vitro* (Figure 7P), and following TGFβ treatment, this was unaffected, whereas expression of IL6 and GADD45B was increased (Figure 7Q and 7R). Silencing of *NFKBIZ* (Figure S7E) led to a suppression of TGFβ-induced IL-6 mRNA and protein expression (Figure 7S and 7T). This was associated with a suppression in *GADD45B* expression (Figure 7U), which lies downstream of IL-6 and is usually induced in response to cellular stress to regulate cell survival and apoptosis (Salvador et al., 2013). Thus, spatial transcriptomic analyses complemented by bioinformatic, tissue, and *in vitro* studies have identified that loss of *NFKBIZ* in alveolar epithelial cells dysregulates the TGFβ/IL-6 axis, which may contribute to the susceptibility of IPF alveolar epithelial cells to environmental stress.

## Discussion

Lung tissue from IPF patients shows marked heterogeneity of pathological changes, which may relate to temporal and/or spatial differences in pathophysiological mechanisms. Here spatial transcriptomics has enabled more precise assessment of the microenvironment and cellular cross-talk in IPF lung tissue through improved understanding of the location of the gene expression signatures detected, identifying discrete changes in homeostatic and pathologic cell populations including loss of endothelial cells, the presence of myofibroblasts throughout IPF lung tissue, as well as the presence of *HAS1*^*hi*^ fibroblasts within fibroblast foci. There is evidence of coordination of multiple cellular compartments contributing to fibrogenesis and aberrant tissue responses through their spatial proximity. Together these data identify potential pathological mechanisms that may occur in early disease including loss of *NFKBIZ* in alveolar epithelial cells.

Application of DSP technology to interrogate the fibrotic niche in human lung fibrosis identified that even morphologically preserved IPF alveolar septae had evidence of dysregulated gene expression and altered cell population proportions. This manifested as an increase in collagen gene expression compared with control alveolar septae, an increase in the proportion of myofibroblasts, the presence of pathologically related epithelial cell populations, which were absent from control lung, as well as a decrease in the proportion of endothelial cells. The decrease in endothelial cells was present in IPF alveolar septae irrespective of whether they were adjacent to fibroblastic foci or more distal and morphologically preserved, suggesting that endothelial dysregulation could be an important contributory factor in the early stages of alveolar dysfunction. Similar to epithelial cells, endothelial cells can undergo endothelial to mesenchymal transition (EndMT) (Jia et al., 2021), a process that has previously been associated with vascular loss and accumulation of mesenchymal cells in IPF (Gaikwad et al., 2020). This hypothesis is supported by our identification of an increase in myofibroblasts in IPF alveolar septae.

Although the decline in the proportion of endothelial cells was not accompanied by an apparent decrease in the number of ATI and ATII alveolar epithelial cells, evidence of epithelial dysregulation was evident. In some alveolar septae adjacent to fibroblastic foci, we observed an increase in transitional ATII cells that have an intermediate phenotype between ATII and ATI cells (Aspal and Zemans, 2020). This enrichment may represent the need for epithelial renewal in IPF alveolar septae. However, we also found an increase in basal cells in alveolar septae closest to fibroblast foci, and as basal cells act as bronchial stem cells in lung injury, their invasion into alveolar epithelium suggests progressive loss of alveolar progenitor cells. This may lead to alveolar remodeling and bronchiolization of the distal lung (Shaykhiev, 2019). Further evidence of perturbation of alveolar epithelial homeostasis within the fibrotic niche was provided by the appearance of *HAS1*^*hi*^ fibroblasts as well as myofibroblasts and loss of lipofibroblast-like PLIN2-positive fibroblasts. In mice, lipofibroblasts support ATII cells, not only by providing lipid substrates for lung surfactant production, but also by maintaining the ATII stem cell niche (Kumar et al., 2014; Liu et al., 2021b; McGowan and Torday, 1997; Xie et al., 2018; Zepp et al., 2017). Thus, our data suggest that loss of alveolar homeostasis involves dysregulation of multiple cell types. As spatially resolved transcriptomic analyses allow the selection of ROIs within IPF lung tissue that are morphologically preserved, this approach can aid identification of early pathogenic mechanisms and provide insight into their temporal relationships.

The apparent inability of immune cells to penetrate the stiffened stroma surrounding fibroblastic foci (Nuovo et al., 2012) is similar to the microenvironment of many solid tumors in which immune cells are excluded by stromal fibrosis. This has been associated with several mechanisms involving mediator-produced CAFs including expression of CXCL12 that mediates T cell exclusion from the tumor and resistance to immunotherapy via CXCR4 (Feig et al., 2013). The importance of this mechanism has been inferred in models of pancreatic and breast cancer in which inhibition of CXCR4 using a clinically approved inhibitor (AMD3100) increased intratumoural T cell accumulation and response to checkpoint inhibition (Chen et al., 2019). Our identification within IPF tissue of high expression of CXCR4 in the immune infiltrate ROIs and the expression of CXCL12 within fibroblastic foci suggests a potentially similar immune exclusion mechanism in lung fibrosis. While AMD3100 has been tested in preclinical models of lung fibrosis, its prophylactic antifibrotic activity was attributed to prevention of CXCL12-mediated migration of circulating fibrocytes into the injured lung.

We identify a significant downregulation of pathways associated with innate immunity in IPF ROIs compared with control lung ROIs, with a marked downregulation of TNFα signaling via NF-κB, downregulation of type I interferon responses, and IL6-related gene expression. The absence of inflammatory gene signatures is consistent with the absence of benefit identified in clinical trials of immune-modulatory drugs in IPF (King et al., 2009; Raghu et al., 2008). Downregulation of interferon-associated gene expression has previously been reported in cells isolated from the peripheral blood of patients with IPF (Huang et al., 2021), and we identified a suppression of type I interferon innate immune responses in IPF lung tissue, confirming through *in vitro* studies that TGFβ was sufficient to suppress type I IFN-regulated gene expression. This observation may be relevant to virus-induced exacerbations (Azadeh et al., 2017). Furthermore, patients with interstitial lung disease have been shown to be at increased risk of death from COVID-19 infection (Drake et al., 2020), and a recent study identified that people with chronic lung disease who are at increased risk of severe COVID-19 infection have evidence of altered immune and inflammatory gene expression, including dysregulation of type I interferon response genes (Bui et al., 2021).

Spatial transcriptomics enabled the identification of a pathway by which loss of *NFKBIZ* in alveolar epithelial cells may perturb the TGFβ/IL-6 axis to dysregulate alveolar homeostasis in IPF. We observed a decrease in IL6-related gene expression, with a significant decrease in *NFKBIZ* and *GADD45B* expression, as well as a decrease in gene sets associated with IL6 signaling and IL6 JAK-STAT signaling. IκBζ, the protein product of *NFKBIZ,* has been identified as a key regulator of IL6 gene expression, and *GADD45B* expression is induced by IL6 signaling (Liebermann and Hoffman, 2002; Yamamoto et al., 2004). We confirmed downregulation of IL6 signaling, as well as the downregulation of *IL6*, *NFKBIZ*, and *GADD45B* in a large-scale transcriptomic study of IPF versus healthy lung tissue (Yang et al., 2013) as well as showing decreased expression of IκBζ in alveolar epithelial cells in IPF lung tissue. We further demonstrated the functional relevance of IκBζ in ATII cells by siRNA knockdown of *NFKBIZ*, which suppressed induction of IL-6 in response to TGFβ. IL-6 is a pleiotropic cytokine that can have either proinflammatory or profibrotic effects. Thus, while blockade of IL-6 signaling attenuates pulmonary fibrosis (Le et al., 2014), IL-6 protects against oxidant-induced death of alveolar epithelial cells, and it regulates surfactant homeostasis in ATII cells (Kida et al., 2005; Matsuzaki et al., 2008). While targeting the IL-6 pathway with monoclonal antibodies such as tocilizumab has provided treatment benefit in a number of diseases including rheumatoid arthritis, its use is associated with an increased risk of non-infectious pulmonary complications, especially interstitial lung disease consistent with an important role for IL-6 in maintaining pulmonary homeostasis (Hadjinicolaou et al., 2011).

In conclusion, spatially resolved deconvolution analyses of diagnostic IPF lung tissue has provided insight into cell composition and microenvironment within and surrounding the fibrotic niche. This approach will support advances in human-disease-relevant models and therapeutic targeting.

### Limitations of the study

It is widely accepted that lung fibrosis involves a complex interplay between multiple cell types including mesenchymal, epithelial, and immune cells, but areas of active fibrogenesis—the fibrotic niche—remain poorly understood. Our study enabled the selection of multiple defined ROIs in spatial proximity within the fibrotic niche: fibroblast foci, adjacent alveolar septae, and immune infiltrates. One limitation of our work is that gene expression profiling was limited to 1,813 genes encompassing cell biology, metabolic, microenvironment, and immune response pathways, so other gene expression profiles are not available. Although our approach enabled robust selection of morphologically distinct regions within the marked heterogeneity of human lung fibrosis tissue, a second limitation is that sampling of additional regions such as small airways was not undertaken and may further inform understanding of fibrogenesis. A third limitation of the study is that the spatial resolution was limited to the defined ROI, so it did not provide RNA profiling at the single cell level (Merritt et al., 2020), with the gene signatures of multiple cell types included within each ROI. We therefore employed spatial deconvolution methodology to further investigate cellular composition. Although these data provide a useful approximation of cell types within ROIs, integration within alternative approaches would be complementary in future work to further investigate the RNA profiles of single cells within the fibrotic niche. Finally, it remains uncertain whether our observations are applicable only to human IPF or also to other forms of progressive lung fibrosis.

## STAR★Methods

### Key resources table

**Table:** 

REAGENT or RESOURCE	SOURCE	IDENTIFIER
**Antibodies**		

Anti-NFKBIZ antibody	Atlas Antibodies	RRID:AB_1854442; Cat#HPA010547
COMP Polyclonal Antibody, Alexa Fluor 555 Conjugated	Stratech	Cat#BS-10286R-A555-BSS
PLOD2-Specific Polyclonal antibody	Proteintech	RRID:AB_10733347; Cat#21214-1-AP
Goat anti-Rabbit IgG (H + L) Highly Cross-Adsorbed Secondary Antibody, Alexa Fluor™ 647	Thermo Fisher Scientific	RRID:AB_2535813; Cat#A-21245
Goat Anti-Rabbit IgG Antibody (H + L), Biotinylated	Vector Laboratories	RRID:AB_2313606; Cat#BA-1000

**Biological samples**		

Lung tissue as reported in experimental model and subject details	University Hospital Southampton	N/A

**Chemicals, peptides, and recombinant proteins**		

DCCM-1 cell culture media	Biological Industries	Cat#K1-0502
Penicillin/streptomycin	Sigma-Aldrich	Cat#P4333
Fetal Bovine Serum	Thermo-Fisher Scientific	Cat#10500-064
L-glutamine	Sigma-Aldrich	Cat#59202C
Haematoxylin 7211	Fisher Scientific	Cat#10034813
Eosin Y	Fisher Scientific	Cat#12677756
Gill’s haematoxylin	Sigma-Aldrich	Cat#GHS132-1L
Proteinase K (20mg/mL)	Life Technologies	Cat#AM2546

**Critical Commercial Assays**		

RNAscope Hiplex v2 assay reagents	ACD Bio	Cat#324445
RNAscope HiPlex Probe - Hs-COMP-T3 - Homo sapiens cartilage oligomeric matrix protein	ACD Bio	Cat#457081-T3
RNAscope HiPlex Probe - Hs-SOD2-T5 - Homo sapiens superoxide dismutase 2 (SOD2) transcript	ACD Bio	Cat#500281-T5
RNAscopeHiPlex Probe - Hs-NFKBIZ-T6 - Homo sapiens NFKB inhibitor zeta (NFKBIZ) transcript	ACD Bio	Cat#497851-T6
RNAscope HiPlex Probe - Hs-COL1A2-T8 - Homo sapiens collagen type I alpha 2 (COL1A2)	ACD Bio	Cat#432721-T8
RNAscope HiPlex Probe - 1Hs-CRABP2-T9Homo sapiens cellular retinoic acid binding protein 2	ACD Bio	Cat#900161-T9
RNAscope® HiPlex Probe - Hs-SFTPC-T11 - Homo sapiens surfactant protein C (SFTPC) transcript	ACD Bio	Cat#452561-T11
GeoMx Cancer Transcriptome Atlas	Nanostring	Cat#GMX-RNA-NGS-CTA-4
GeoMx Solid Tumor TME Morphology Kit, Human RNA Compatible	Nanostring	Cat#GMX-RNA-MORPH-HST-12
GeoMx RNA Slide Prep Kit for FFPE	Nanostring	Cat#GMX-PREP-RNA-FFPE-12
GeoMx Seq Code Pack: A & B	Nanostring	Cat#GMX-NGS-SEQ-AB
Human IL-6 Quantikine ELISA Kit Human IL-6 Quantikine ELISA Kit	RnD Systems	RRID:AB_2893335 Cat#D6050

**Deposited Data**		

Single-cell RNA-sequencing reveals profibrotic roles of distinct epithelial and mesenchymal lineages in pulmonary fibrosis	NCBI Gene Expression Omnibus	GSE135893
Molecular phenotyping of the idiopathic interstitial pneumonias	NCBI Gene Expression Omnibus	GSE32537
Spatial transcriptome profiling identifies CREB1 as a regulator of core transcriptional programs in idiopathic pulmonary fibrosis	NCBI Gene Expression Omnibus	GSE169500

**Experimental Models: Cell Lines**		

ATII^ER:KRASV12^ cell line	Gift of Julian Downward, Francis Crick Institute, London (Cancer Discov. 2013; 3:548–63. Immunity. 2017; 47:1083–99. Cell Death and Differentiation, 2019; 26:943-057)	N/A

**Oligonucleotides**		

ON-TARGETplus siRNA SMARTpool	Dharmacon	Cat#L-013497-00-0010
NFKBIZ gene expression assay	Thermo Fisher Scientifc	Cat#Hs00230071
IL6 gene expression assay	Thermo Fisher Scientifc	Cat#Hs00174131_m1
GADD45B gene expression assay	Thermo Fisher Scientifc	Cat#Hs00169587_m1

**Software and Algorithms**		

R	The R software Foundation	R version 4.0.2
Prism	Graphpad	Version 9.0.1
Hiplex Image Registration Software	ACD Bio	N/A

### Resource availability

#### Lead contact

Further information and requests for resources and reagents should be directed to and will be fulfilled by the lead contact, Dr. Mark Jones, mark.jones@soton.ac.uk.

#### Materials availability

This study did not generate new unique reagents.

#### Data and code availability

All data generated during this study are included in the manuscript and supporting files. This paper also analyzes existing, publicly available data. The accession numbers for the datasets are listed in the key resources table. The public processed IPF single-cell data from Habermann et al. (GSE135893) can also be accessed via the IPF cell atlas portal at http://www.ipfcellatlas.com. The public processed healthy lung single-cell data for NFKBIZ expression can be accessed via the Human Protein Atlas, v21.proteinatlas.org: https://www.proteinatlas.org/ENSG00000144802-NFKBIZ/celltype/lung. The public healthy lung tissue staining for IκBζ can be accessed via the Human Protein Atlas, v21.proteinatlas.org: https://www.proteinatlas.org/ENSG00000144802-NFKBIZ/tissue/lung#.
All original code is available in this paper’s supplemental information in supporting file 1. Any additional information required to reanalyze the data
reported in this paper is available from the lead contact upon request.

### Experimental model and subject details

Clinically indicated diagnostic surgical lung biopsy specimens were from patients with a subsequent multidisciplinary diagnosis of IPF (n = 3) according to international consensus guidelines (Raghu et al., 2018). Specimens had been diagnosed as showing a typical usual interstitial pneumonia pattern by 2 independent histopathologists. Control lung tissue was from macroscopically normal lung (n = 3) sampled remote from the cancer site in age and sex-matched patients undergoing lobectomy surgery for early-stage lung cancer. Details of the donor characteristics including age, gender, and disease status are provided in Table S4B. Formalin fixed paraffin embedded tissue blocks had received standard processing with fixation in neutral buffered formalin for 48 h and embedding in paraffin wax and tissue blocks had been archived for less than seven years. The study was approved by the Mid and South Buckinghamshire Local Research Ethics Committee (ref 07/H0607/73), and all subjects gave written informed consent.

The human alveolar type 2 cell line (ATII^ER:KRASV12^) was cultured in DCCM-1 (Biological Industries Ltd.) supplemented with 10% FBS (Life Technologies), 1% penicillin, 1% streptomycin and 1% L-glutamine (all from Sigma Aldrich), as previously described (Yao et al., 2019, 2021). No mycoplasma contamination was detected in the cell lines used.

### Method details

#### Haematoxylin and eosin (H&E) staining

Tissue sections (5 μm) were dewaxed by immersing in xylene for 2 × 4 min and rehydrated by 2 changes in 100% ethanol followed by one wash in 95% ethanol for 20 s each before placing in running tap water for 1 min. Tissues were stained with Haematoxylin 7211 (Fisher Scientific, #10034813) and counterstained with Eosin Y (Fisher Scientific, #12677756) for 20 s. As the DSP protocol is non-destructive, tissue sections can be stained with H&E once the DSP protocol is complete by placing sections in dH_2_O before immersing slides in Haematoxylin and following the remaining protocol as standard.

#### IκBζ immunohistochemistry

Control or IPF lung tissues (n = 3 donors) were fixed and embedded in paraffin wax; tissue sections (4 μm) were processed and stained as previously described (Brereton et al., 2022; Yao et al., 2019). Briefly, the tissue sections were de-waxed, rehydrated and incubated with 3% hydrogen peroxide in methanol for 10 min to block endogenous peroxidase activity before antigen retrieval (microwave EDTA pH 8). Sections were incubated at room temperature with a primary antibody against IκBζ (1:50, HPA010547, Sigma-Aldrich), followed by a biotinylated secondary antibody (1:500, Vector Laboratories Ltd., UK); antibody binding was detected using streptavidin-conjugated horseradish peroxidase and visualised using DAB before counter-staining with Gill’s Haematoxylin. Images were acquired using an Olympus VS110 digital slide scanner.

#### Immunofluoresence

Formalin fixed, paraffin embedded (FFPE) IPF and healthy lung tissue tissue was deparaffinised before antigen retrieval (microwave citrate buffer pH 6). Tissue was incubated overnight at 4°C with an Alexa Fluor 555 conjugated anti-COMP Polyclonal Antibody, (1:100, Stratech) and a PLOD2-Specific Polyclonal antibody (1:100, Proteintech) before incubation with an Alexa Fluor 647 conjugated secondary antibody (1:250, Thermo Fisher Scientific) for 2 h at room temperature before a 15 min incubation with DAPI (1:500). Confocal images of representative ROIs were taken using a Leica TCS-SP8 confocal system on a Leica DMI8 inverted microscope stand.

#### RNAscope HiPlex in situ hybridisation

RNAscope was performed according to the manufacturer’s instructions using the following probes and reagents. Briefly, FFPE IPF and healthy lung tissue sections were deparaffinised, before fluorescently conjugated RNAscope hiplex probes (ACD Bio) against *COMP, COL1A2, CRABP2, SFTPC, SOD2* and *NFKBIZ* were hybridised and signal amplified using RNAscope Hiplex V2 (488, 550,650) reagents over multiple rounds of probe hybridisation and strippings. Confocal images of representative ROIs were taken using a Leica TCS-SP8 confocal system with ROI positions stored for relocation over multiple rounds of hybridisation, stripping and imaging and background fluorescence removed using a reference image of the same ROI without probes using RNAscope hiplex image registration software (ACD Bio).

#### Spatial transcriptomics

##### GeoMx slide processing

For the Nanostring GeoMx CTA assay, slides were prepared according to the Leica Biosystems BOND RX FFPE RNA Slide Preparation Protocol in the GeoMx – NGS Slide Preparation User Manual (NanoString, MAN-10115-04). Morphology marker solution was created using SYTO13 (Nanostring), Pan-Cytokeratin morphology marker (Nanostring), and CD45 morphology marker (Nanostring).

##### GeoMx DSP instrument and ROI selection

Slides were loaded into the slide holder of the GeoMx DSP instrument and covered with 2 mL of buffer S. Each slide was scanned with a 20× objective with the scan parameters: 50 ms FITC/525 nm, 300 ms Cy3/568 nm, 300 ms Texas Red/615 nm. A serial section next to the one used for DSP analysis was stained with H&E and potential ROIs identified. Corresponding regions of interest (ROIs) were then selected on the DSP slide, with regions of interest identified by their morphology using pan-cytokeratin as an epithelial marker and CD45 as an immune marker. In IPF lung tissue selected ROIs were fibroblast foci (IPF-FF) and their adjacent alveolar septae (IPF-AAS), nearby immune infiltrates identified by CD45+ staining (IPF-IM), morphologically preserved alveolar septae distant from fibroblast foci (IPF-DAS), and blood vessel walls containing smooth muscle (IPF-BV). In healthy control lung tissue selected ROIs were alveolar septae (CTRL-AS) and blood vessel walls containing smooth muscle (CTRL-BV).

After the DSP protocol was complete the slides were also stained with H&E and each section was examined and ROI annotation confirmed by a lung histopathologist (A.F.). After ROIs were approved, the GeoMx DSP exposes the selected regions to UV light which photocleaves the UV cleavable barcode linked from the bound RNA probes, which are collected and deposed into separate wells in the DSP collection plate.

##### GeoMx RNA illumina library preparation

DSP collection sample plates were dried, resuspended in nuclease-free water, and amplified using PCR according to the manufacturer’s protocol.

Following PCR amplification, the indexed libraries were pooled, harvested and washed using AMPure XP beads (Beckman Coulter) prior to elution. Sequencing library size was assessed with a High Sensitivity D1000 Screen Tape assay for TapeStation systems (Agilent Technologies) and the expected library size of ∼150 bp was observed. Purified libraries were sequenced by Illumina NovaSeq SP (2x 50bp).

##### Data processing and QC

The FASTQ reads from sequenced DSP library were processed by the GeoMx NGS Pipeline to convert sequencing reads into ROI counts (Nanostring, MAN-10118-03). After processing, counts were uploaded to the GeoMx DSP Data Analysis Suite (NanoString). QC steps were carried out to assess raw read threshold, percent aligned reads and sequencing saturation. The limit of quantification (LOQ) was determined as the negative probe geomean + 2x the geometric standard deviations of the negative probes. Any probes that could not be detected in at least 5% of ROIs were filtered, leaving 1086 genes from the original 1811. Raw counts were then normalized by Q3/upper quartile normalization (Bullard et al., 2010). Normalized counts were exported and processed further using R.

##### Data analysis

Data analysis was performed in R (R Core Team, 2020). Graphics were produced using ggplot2 unless otherwise stated (Wickham, 2016, p. 2).

##### T-stochastic nearest neighbor embedding

The R package Rtsne (Krijthe, 2021) was used to generate T stochastic nearest neighbor embedding (T-SNE) embeddings. These were then plotted using ggplot2 to show how different regions of interest cluster on a dimensional reduction plot.

##### Pearson correlation heatmap

Pearson correlation coefficients between each ROI were generated and plotted using the pheatmap R package (Kolde, 2019).

##### Differentially expressed genes

A Wilcoxon rank-sum test was used to identify differentially expressed genes between different regions of interest. p values were adjusted using Benjamini-Hochberg multiple test correction. An adjusted p value cut-off of 0.05 and a logFC of 0.25 was used to identify differentially expressed genes. Variable genes across the whole dataset were identified by a Kruskal-Wallis test (p < 0.05) across the dataset in order to show clustering of different ROI groups by heatmap.

##### Gene expression heatmaps

Heatmaps were produced using the heatmap.2() function from the R package gplots (Galili, 2021). Heatmaps used by-row scaling, and ROIs were clustered using the default hierarchical clustering algorithm.

##### Gene set variation analysis

Gene set variation analysis was performed in R using the R package GSVA (Hänzelmann et al., 2013). The gsva() function was used to assess the enrichment of gene sets in each ROI. A Wilcoxon rank-sum test with Benjamini-Hochberg multiple test correction (adjusted p value <0.05) was then used to identify groups of ROIs with significantly enriched GSVA scores. TGFβ signaling genes used were ACTA2, COL1A1, COL3A1, CCN2, IL11, CDH2. Additional gene sets are taken from the gene ontology consortium or Molecular Signatures database Hallmark gene sets. Gene sets used in this comparison are in Table S4C.

##### Enrichment plots

To generate enrichment scores used for the pathway bubble plots each gene was assigned to a particular signaling pathway, immune regulator, metabolic or cell process according to the gene target group memberships provided by NanoString (https://nanostring.com/products/geomx-digital-spatial-profiler/geomx-rna-assays/geomx-cancer-transcriptome-atlas/). Each gene was assigned to a single group and typically the first target group listed for each gene was used. A full list of target group assignments for genes is available in Table S3. For each gene set, the mean normalized counts for all genes within that gene set in each region were taken. Mean counts were then scaled by taking the Z-Scores for all regions for each gene set. Bubble plots were then made using the ggplot2 package.

##### Cell profile matrix

The cell profile matrix was generated using a single cell RNA sequencing dataset of cells taken from different ILDs (interstitial lung diseases), generated by Habermann et al. (Habermann et al., 2020). Cells originating from IPF tissue and normal lung tissue were extracted, and the R package Seurat was used to generate a normalised genes x cells matrix (Hao et al., 2021). The FindAllMarkers() function in Seurat was then used to identify marker genes associated with the different types of cells identified in this study. This uses a Wilcoxon rank-sum test to identify differentially expressed genes associated with different cell types. A p value of <0.05 and a logFC of >2 was used to identify strongly differentially expressed marker genes. The mean expression of these marker genes was calculated, and the marker genes were cross-referenced with the GeoMX cancer transcriptomics atlas (CTA) to ensure that marker genes for each cell type were present in the CTA. This set of marker genes was then used as the cell profile matrix for spatial and reverse deconvolution.

##### Spatial deconvolution

Spatial deconvolution was performed using the SpatialDecon R package(Danaher et al., 2020). The spatialdecon() function was used to deconvolute the bulk gene expression data from individual regions of interest into the proportions of different cell types associated with each ROI. Normalised gene expression values from the GeoMX DSP were input as the expression matrix, and the cell profile matrix described above was used for the markers. Background values were derived from the negative probe expression values. With these inputs, the spatialdecon() function outputs the relative proportions of each cell type identified in the cell profile matrix.

##### Ligand-receptor analyses

Potential Ligand-receptor interactions were determined using the R package NicheNet (Browaeys et al., 2020). Gene counts were thresholded such that a gene was considered not to be expressed within a given ROI if it was below the negative control probe counts and all genes present in the DSP dataset were used as background genes. NicheNet infers ligand-receptor interactions by determining ligands expressed in sender regions (Immune and adjacent alveolar ROIs), receptors expressed in receiver regions (fibroblastic foci), and signaling within receiver regions. The ligand-receptor pairs most likely to induce expression of a fibrotic foci specific genes were then determined using a Pearson score cut-off of 0.1. NicheNet results were then cross referenced with results obtained using the Celllinker web server (Zhang et al., 2021), which predicts all Ligand/Receptor pairs based on the presence of these ligands and receptors within populations. Thresholded ROI counts were also used for this analysis and a gene was considered to be expressed in a particular type of ROI if it was above the threshold in 25% of ROIs. A p-Value cut-off of 0.05 was used with 100 statistical iterations.

##### Type 2 alveolar cell *in vitro* studies

ATII^ER:KRASV12^ cells were transfected with the indicated siRNA oligos at a final concentration of 100 nM using RNAiMax Lipofectamine reagent (Life Technologies). After 48 h, cells were treated with TGFβ at 0, 1, or 5 ng/mL for a further 24 h prior to harvesting for analysis of mRNA expression and protein release. TGFβ1 was from PeproTech. Short interfering RNA (siRNA) oligos against *NKFBIZ* (On-target Plus *NFKBIZ* Smartpool) were purchased from Dharmacon. Sequences are available from Dharmacon, or on request. As a negative control, we used On-target Plus Non Targeting Pool siRNA (Dharmacon).

For assessment of innate immune responses, cells were treated with synthetic double-stranded RNA (Polyinosinic:polycytidylic acid (poly I:C) (Invivogen) at 1 or 5 μg/mL for 24 h in the absence or presence of TGFβ (1 or 5 ng/mL). Cells were harvested for mRNA expression analysis by RT-qPCR.

### Quantification and statistical analysis

#### Statistical details can also be found in the figure legends

The details of the analysis can be found in supplementary code.

All statistical tests and graphical depictions of results were performed using R version 4.0.2 (R software Foundation), or GraphPad Prism version 9.0.1 software (GraphPad Software, La Jolla, CA). For all tests, p < 0.05 was considered statistically significant. Statistical significance on figures and supplemental figures is labeled as follow: ^∗^p < 0.05, ^∗∗^p < 0.01, ^∗∗∗^p < 0.001, ^∗∗∗∗^p < 0.0001.

## Supplemental information

Document S1. Figures S1–S7

Table S1. Normalized digital spatial profiling data, related to Figures 2–7 Normalized gene expression data for individual regions of interest using the NanoString GeoMx CTA panel. Any probes that could not be detected in at least 5% of ROIs were filtered, leaving 1,086 genes from the original 1,811. Raw counts were then normalized by Q3 normalization. 60 regions of interest; control alveolar septae (n=12), IPF distant alveolar septae (n=10), IPF adjacent alveolar septae (n=10), IPF fibroblastic foci (n=10), IPF immune infiltrates (n=6), IPF blood vessels (n=6), and control blood vessels (n=6).

Table S2. Metadata for digital spatial profiling data, related to Figure 2 Metadata includes unique ROI identifiers, sample descriptors, and colors used for heatmap generation.

Table S3. Target group assignments for genes used to generate enrichment plots shown in Figure 2 The median Z score for all genes present in each group was used to generate the bubble plots in Figure 2.

Table S4. related to Figures 2, 4, 5, and 7 (A) Differentially expressed genes between IPF adjacent alveolar septae and healthy alveolar septae ROI. (B) Tissue donor demographics. (C) Gene sets used for gene set variation analysis.

Supplementary File 1. R script used to perform analysis of digital spatial profiling data and generate figures, related to Figures 2–7

Document S2. Article plus supplemental information
