# Supplementary material for: Spatially resolved deconvolution of the fibrotic niche in lung fibrosis
Source: Cell Rep. 2022 Aug 16;40(7):111230. doi: 10.1016/j.celrep.2022.111230 (PMC10073410; doi:10.1016/j.celrep.2022.111230)
Supplement: Document S1. Figures S1–S7 [file mmc1.pdf]

Cell Reports, Volume *40*

Supplemental information

Spatially resolved deconvolution of the fibrotic niche in lung fibrosis

Michael Eyres, Joseph A. Bell, Elizabeth R. Davies, Aurelie Fabre, Aiman Alzetani, Sanjay Jogai, Ben G. Marshall, David A. Johnston, Zijian Xu, Sophie V. Fletcher, Yihua Wang, Gayle Marshall, Donna E. Davies, Emily Offer, and Mark G. Jones

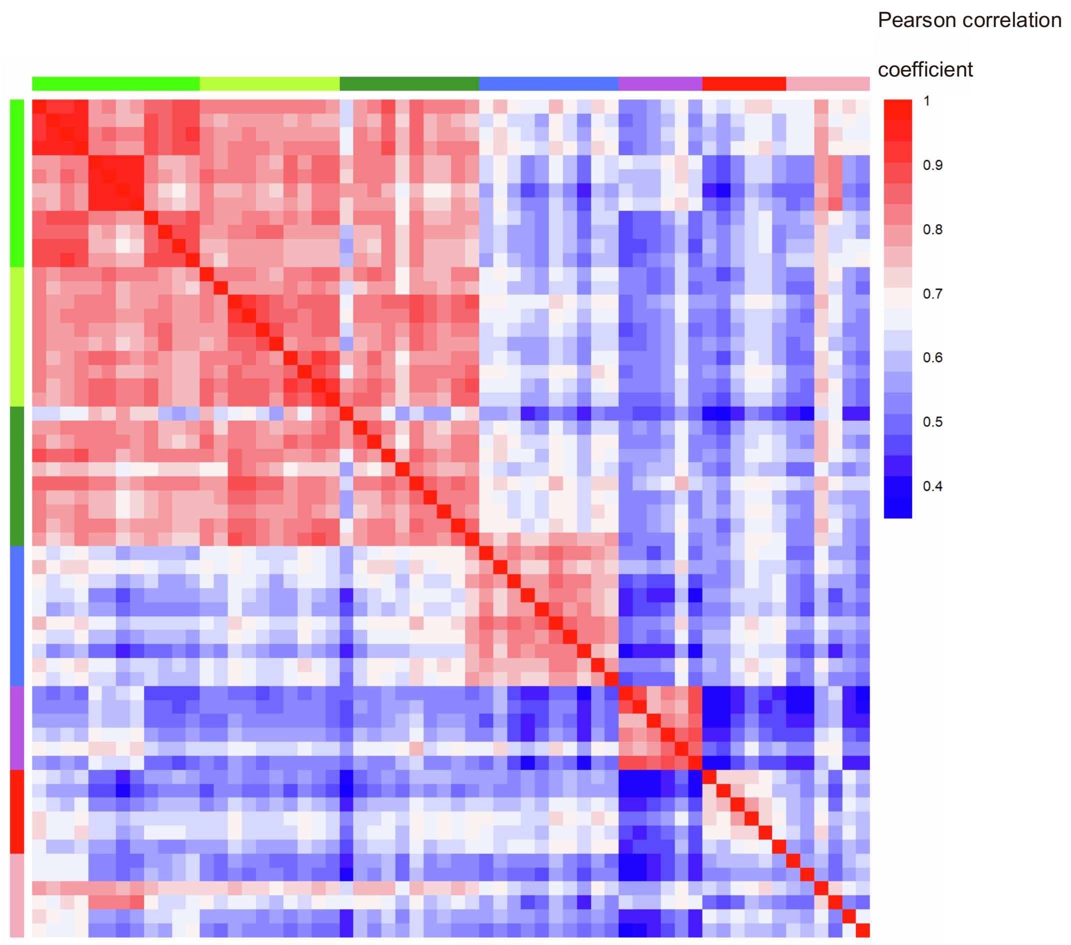

Figure S1. Pearson correlation heatmap. Related to Figure 2. Digital spatial profiling of 60 regions of interest (control alveolar septae, IPF distant alveolar septae, IPF adjacent alveolar septae, IPF fibroblastic foci, IPF immune infiltrates, IPF blood vessels, and control blood vessels) was performed. Heatmap identifies pearson correlation coefficients between different ROIs.

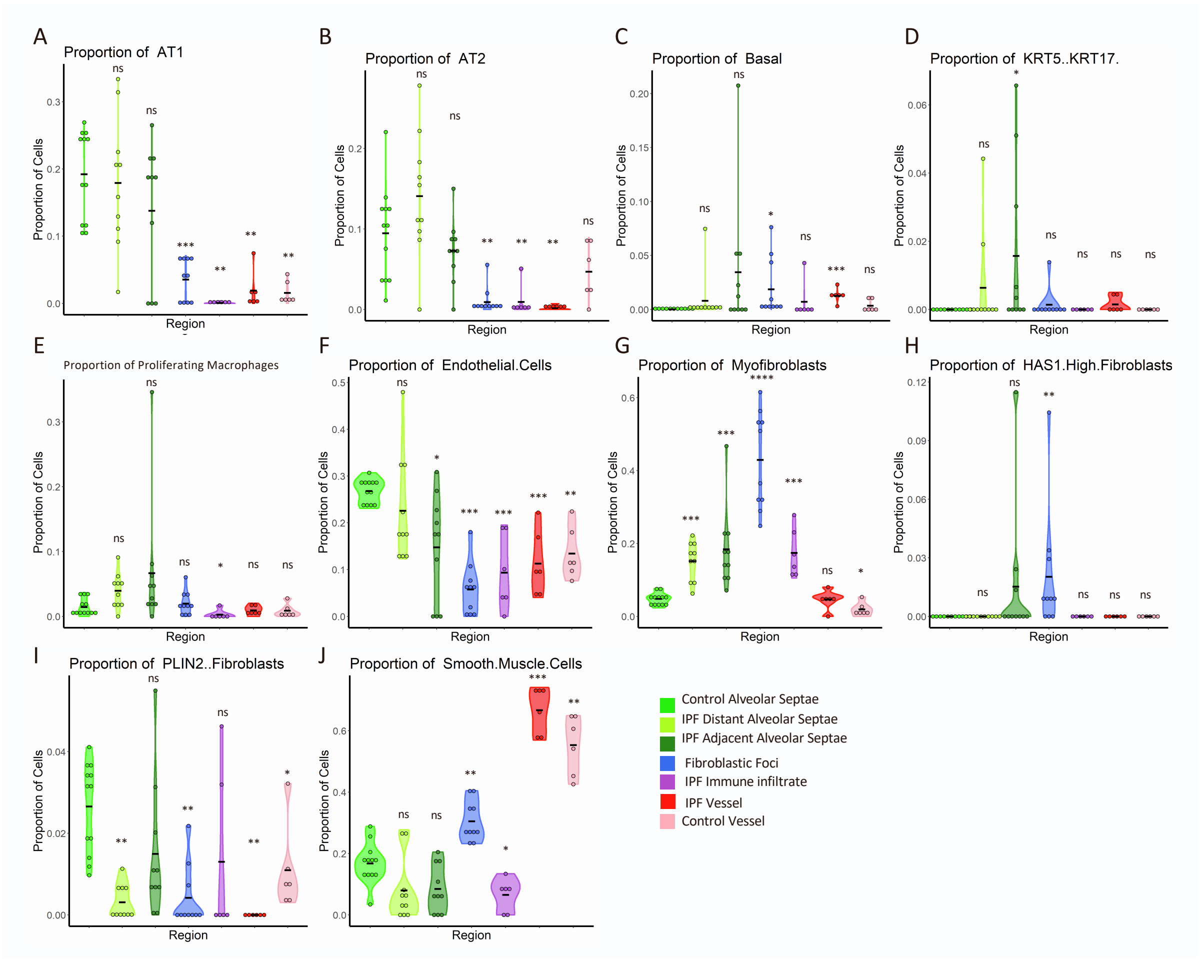

Figure S2. Proportion of different cell types from spatial deconvolution data. Related to Figure 3. Digital spatial profiling of 60 regions of interest (control alveolar septae, IPF distant alveolar septae, IPF adjacent alveolar septae, IPF fibroblastic foci, IPF immune infiltrates, IPF blood vessels, and control blood vessels) was performed followed by spatial deconvolution based on a signature matrix derived from the single-cell RNAseq found in Habermann et al. (A-L). Violin plots showing proportions of different cell types by individual ROI according to spatial deconvolution analysis. (A) Type 1 alveolar cells, (B) Type 2 alveolar cells, (C) Basal Cells, (D) $KRT5^{-}$/$KRT17^{+}$ cells, (E) proliferating macrophages, (F) endothelial cells, (G) myofbroblasts, (H) $\mathit{HAS1}^{hi}$ fibroblasts, (I) PLIN2+ fibroblasts, (J) Smooth muscle cells. Statistical comparisons are relative to control alveolar septae. *p < 0.05, **p < 0.01, ***p < 0.001, ****p<0.0001 by Wilcoxon test with Benjamini-Hochberg multiple test correction.

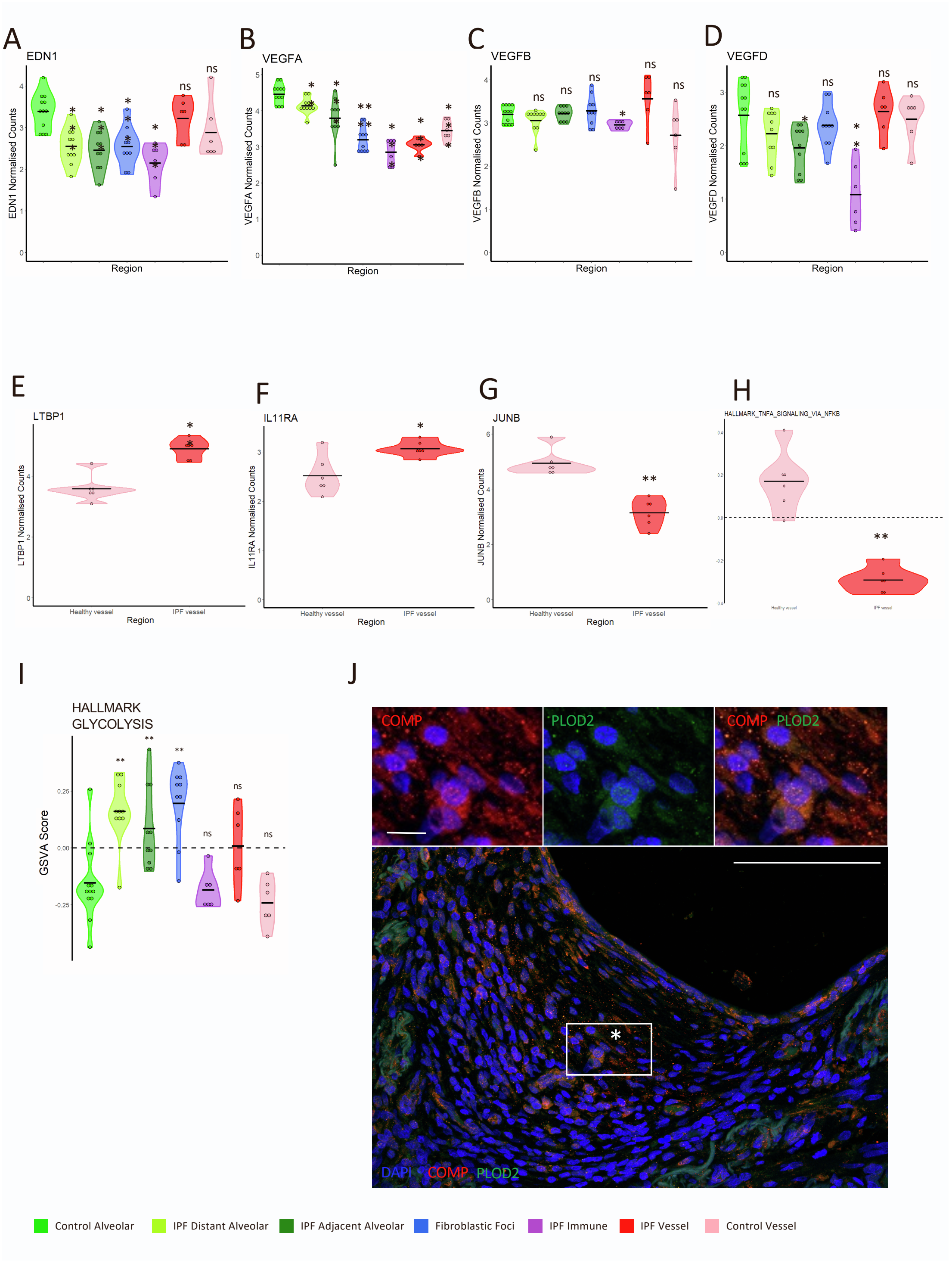

Figure S3. Expression and immunofluorescence of genes/proteins of interest. Related to Figure 4. Digital spatial profiling of 60 regions of interest (control alveolar septae, IPF distant alveolar septae, IPF adjacent alveolar septae, IPF fibroblastic foci, IPF immune infiltrates, IPF blood vessels, and control blood vessels) was performed. Violin plots showing gene expression within ROIs as indicated of (A) *EDN1*, (B) *VEGFA*, (C) *VEGFB*, (D) *VEGFD*. (A-C). Gene expression of (E) *LTBP1*, (F) *IL11RA*, (G) *JUNB* in IPF and control blood vessels. (H). Gene set variation analysis of the TNFα signalling via NFκB Hallmark gene set. (I) Gene set variation analysis of the Hallmark Glycolysis gene set. Statistical comparisons are relative to control alveolar septae (A-D), control blood vessel (E-H), or IPF fibroblastic foci (I). *$p < 0.05$, **$p < 0.01$, ***$p < 0.001$, ****$p<0.0001$ by Wilcoxon test. (J) Representative Immunostaining for COMP and PLOD2 with a fibroblastic focus identified by *. Scale bar is 100μm. Inset scale bar is 10μm.

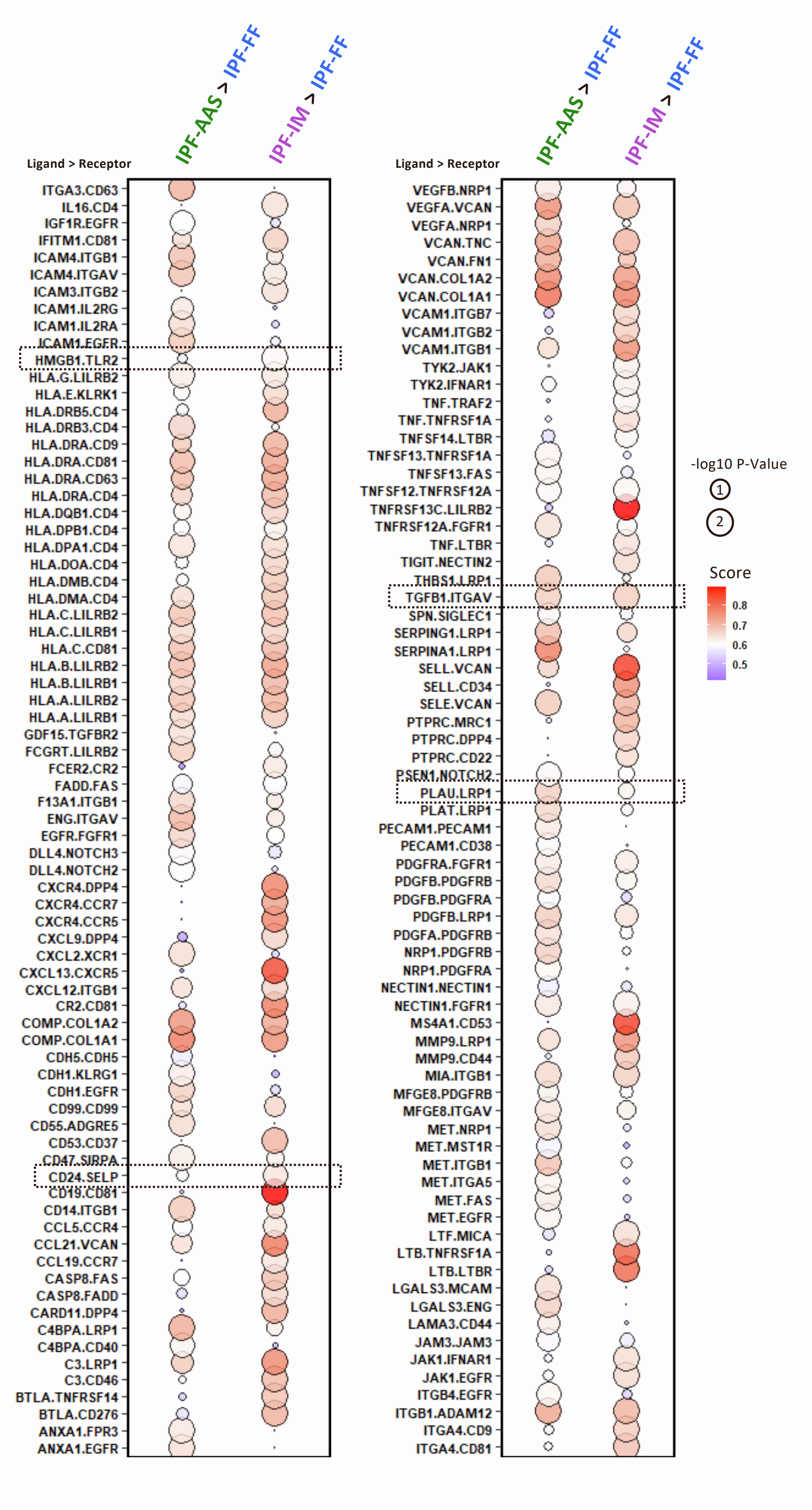

Figure S4. List of selected putative ligand-receptor pairs involved in cross-talk in sender regions (Immune (IPF-IM) or adjacent alveolar (IPF-AAS) ROIs) and receptors expressed in receiver regions (fibroblastic foci (IPF-FF)). Related to Figure 5. The list was generated by in silico ligand receptor analysis of genes using Cellinker.

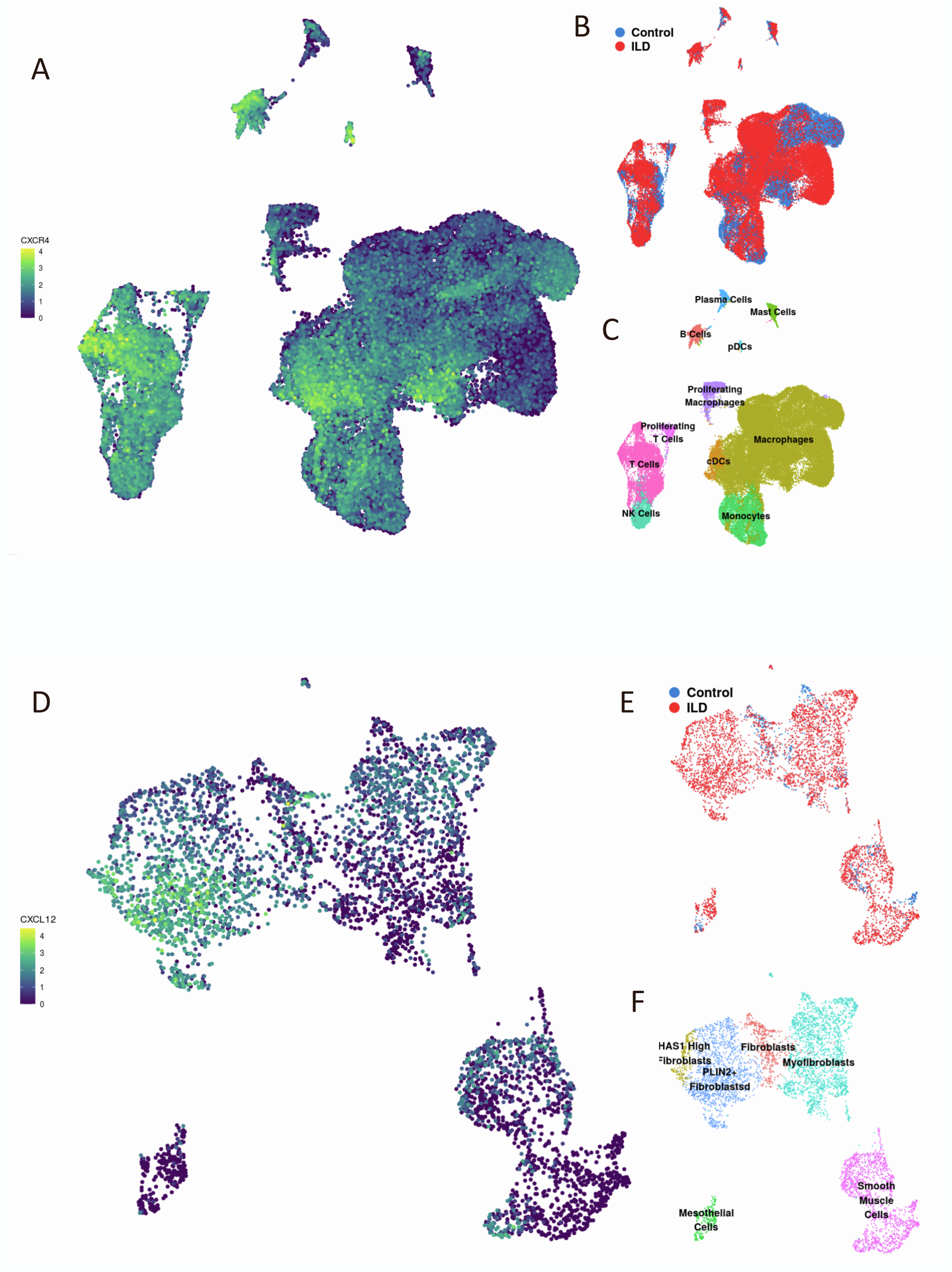

Figure S5. Gene expression of CXCR4 and CXCL12 within a single-cell RNA sequencing dataset. Related to Figure 6. Dataset GSE135893 was accessed via http://www.ipfcellatlas.com (A) CXCR4 expression within immune cell populations with corresponding t-SNE plot of single cell immune cell populations showing diagnosis of the patients of origin for each immune cell (B) and clustering of different immune cell types (C). (D) CXCL12 expression within mesenchymal cell populations with corresponding t-SNE plot of single cell mesenchymal cell populations showing diagnosis of the patients of origin for each mesenchymal cell (E) and clustering of different lung mesenchymal types (F). Image credit: ipfcellatlas.com.

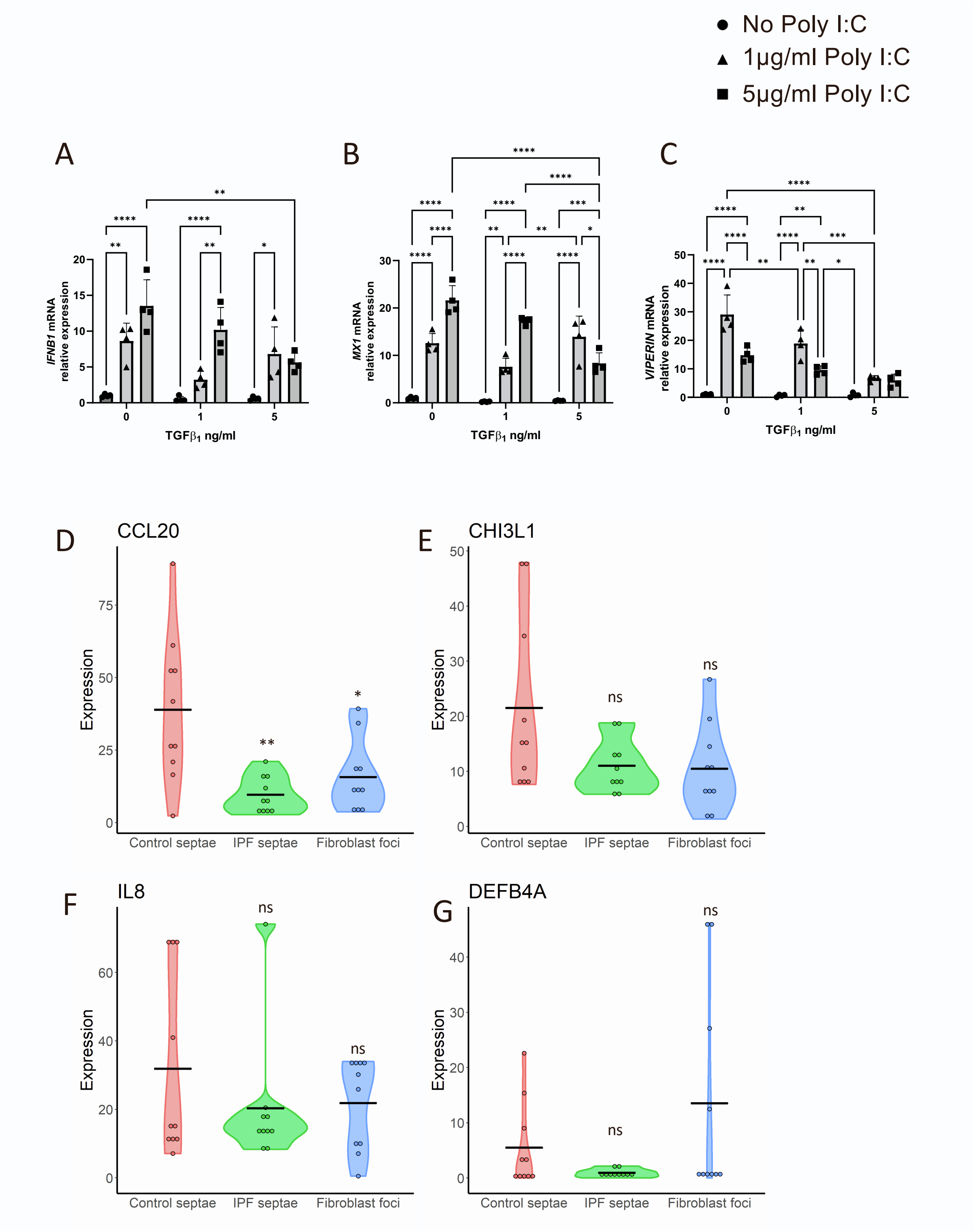

Figure S6. Suppression of Type 1 interferon response genes in alveolar type II epithelial cells following TGF-β treatment and expression of IL6 pathway genes in GSE169500. Related to Figure 7. (A-C) Type 2 alveolar epithelial cells were treated with the viral mimetic Polyinosinic:polycytidylic acid (poly I:C) at the concentrations indicated and treated with TGF-β as shown. Relative gene expression of *IFNB1* (A), *MX1* (B) and *VIPERIN* (C) was determined by qRT-PCR and data analysed using the ΔΔCt method. Data are mean ± SD; n = 4 across 2 independent experiments.*$p < 0.05$, **$p < 0.01$, ***$p < 0.001$ by 2-way ANOVA with Tukey's multiple comparison test. (D-G) Violin plots of expression of (D) *CCL20*, (E) *CHI3L1*, (F) *IL8*, and (G) *DEFB4A* within control alveolar septae, IPF alveolar septae, and fibroblast foci (n=10 individual healthy and IPF donors). Relative expression levels are calculated as Fragments Per Kilobase of transcript per Million mapped reads (FPKM). Data accessible from GSE169500. Comparisons are relative to Control Septae *$p < 0.05$, **$p < 0.01$, ***$p < 0.001$, ****$p<0.0001$ by Wilcoxon test with Benjamini Hochberg multiple test correction.

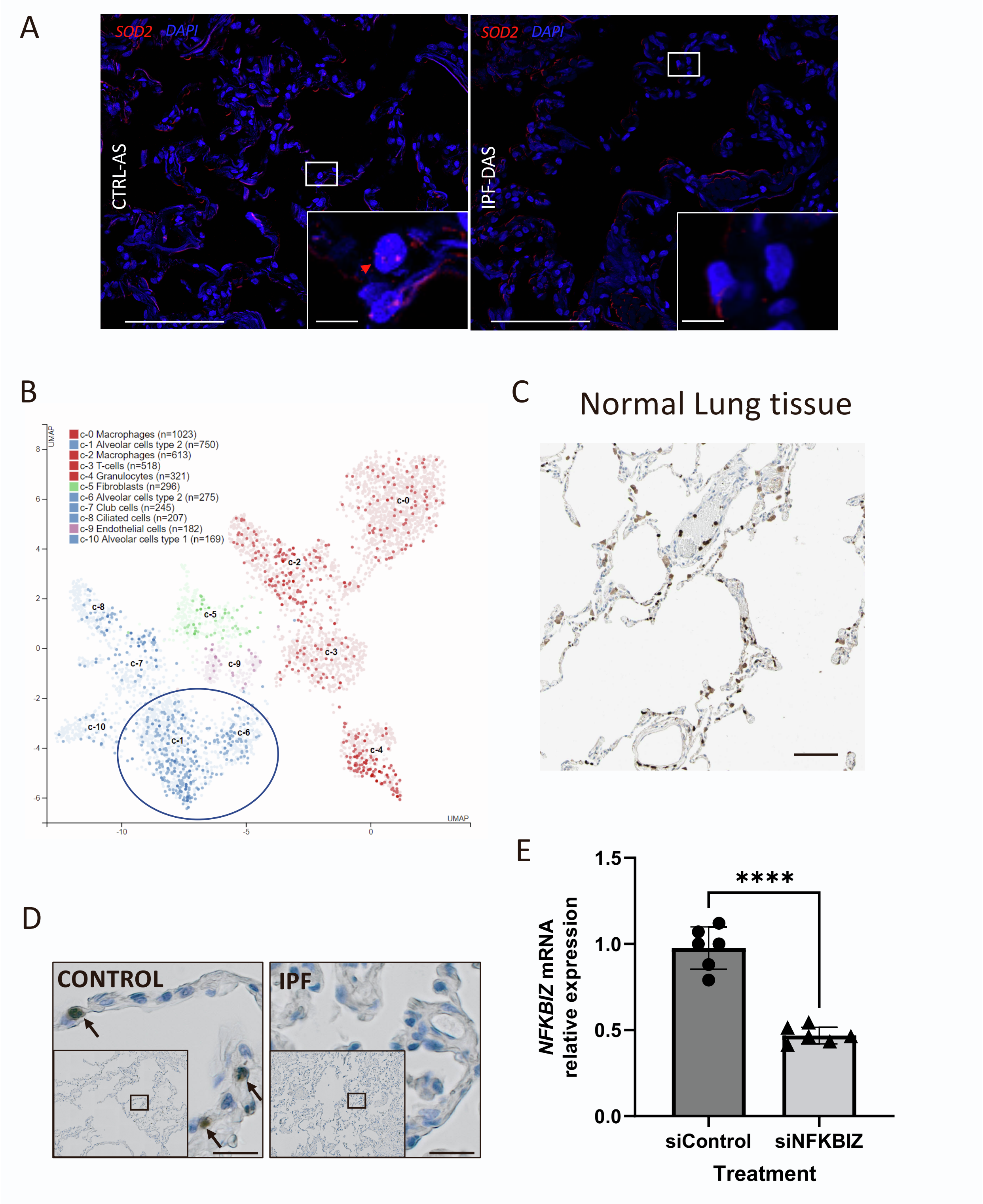

Figure S7. *SOD2* RNA in situ hybridization, IκBζ staining and *NFKBIZ* expression. Related to Figure 7. (A) Representative multiplexed RNA *in situ* hybridization for *SOD2* expression in control alveolar septae and IPF distal alveolar septae (n=3 per group). Scale bars are 100um and inset scale bars are 10um (B) UMAP plot showing *NFKBIZ* expression in single cell lung RNAseq data. Image credit: Human protein atlas, image available from v21.proteinatlas.org: https://www.proteinatlas.org/ENSG00000144802-NFKBIZ/celltype/lung. (C) IκBζ staining in human lung tissue Image credit: Human Protein Atlas, image available from v21.proteinatlas.org: https://www.proteinatlas.org/ENSG00000144802-NFKBIZ/tissue/lung#. Scale Bar is 100 um. (D) Representative immunohistochemical staining of control and IPF lung tissue (n=3 per group) for IκBζ using DAB (brown). Arrows identify representative IκBζ staining. Scale bar is 20 µm. (E) Relative expression

of *NFKBIZ* in Type 2 alveolar epithelial cells transfected with *NFKBIZ* targeting siRNA or control siRNA for 48hrs. After a further 24hrs, relative gene expression was determined by *qRT-PCR* and analysed using the ΔΔCt method. Data are mean ± SD; n = 6 across 3 independent experiments.*$p < 0.05$, **$p < 0.01$, ***$p < 0.001$, ****$p<0.0001$ by T test with Welch's correction.
